# Blood-brain barrier dysfunction and Alzheimer’s disease: associations, pathogenic mechanisms, and therapeutic potential

**DOI:** 10.3389/fnagi.2023.1258640

**Published:** 2023-11-13

**Authors:** Yanting Chen, Yanfang He, Jinling Han, Wenyan Wei, Feng Chen

**Affiliations:** ^1^Department of Neurology, Shenzhen Sixth People’s Hospital, Huazhong University of Science and Technology Union Shenzhen Hospital, Shenzhen, China; ^2^Department of Neurology, Guangdong Key Laboratory of Age-Related Cardiac and Cerebral Diseases, Affiliated Hospital of Guangdong Medical University, Zhanjiang, China; ^3^Department of Gerontology, Affiliated Hospital of Guangdong Medical University, Zhanjiang, China; ^4^Key Laboratory of Animal Models and Human Disease Mechanisms of the Chinese Academy of Sciences & Yunnan Province, Kunming Institute of Zoology, Chinese Academy of Sciences, Kunming, Yunnan, China; ^5^Department of Intensive Care Medicine, Affiliated Hospital of Guangdong Medical University, Zhanjiang, China

**Keywords:** Alzheimer’s disease, blood-brain barrier, neuroinflammation, apolipoprotein E, aging

## Abstract

Alzheimer’s disease (AD) is a common neurodegenerative disorder characterized by the accumulation of amyloid-beta (Aβ), hyperphosphorylation of tau, and neuroinflammation in the brain. The blood–brain barrier (BBB) limits solutes from circulating blood from entering the brain, which is essential for neuronal functioning. Focusing on BBB function is important for the early detection of AD and in-depth study of AD pathogenic mechanisms. However, the mechanism of BBB alteration in AD is still unclear, which hinders further research on therapeutics that target the BBB to delay the progression of AD. The exact timing of the vascular abnormalities in AD and the complex cause-and-effect relationships remain uncertain. Thus, it is necessary to summarize and emphasize this process. First, in this review, the current evidence for BBB dysfunction in AD is summarized. Then, the interrelationships and pathogenic mechanisms between BBB dysfunction and the risk factors for AD, such as Aβ, tau, neuroinflammation, apolipoprotein E (ApoE) genotype and aging, were analyzed. Finally, we discuss the current status and future directions of therapeutic AD strategies targeting the BBB. We hope that these summaries or reviews will allow readers to better understand the relationship between the BBB and AD.

## 1. Introduction

Currently, there are more than 50 million dementia patients worldwide, with Alzheimer’s disease (AD) accounting for approximately 60–80% (Author, 2022). AD is a progressive and irreversible neurodegenerative disease, and its clinical manifestations include memory loss, cognitive dysfunction, and behavioral disturbances (Frozza et al., 2018). The pathological features of AD include Aβ accumulation, abnormal tau protein metabolism, and a neuroinflammatory response (Breijyeh and Karaman, 2020). Recent autopsy studies have shown that dysfunction of the blood–brain barrier (BBB) is closely associated with AD (Kurz et al., 2022). Disruption of BBB integrity in AD was found to precede symptoms of cognitive impairment by several years, with brain capillary damage and BBB disruption in the hippocampus considered early biomarkers of cognitive dysfunction (Nation et al., 2019). Disruption of the BBB results in nonselective entry of solutes from circulating blood into the extracellular fluid of the central nervous system (CNS), where the neurons are located, further damaging the affected brain tissue and triggering neurodegeneration (Zhao et al., 2015). BBB disruption is increased in AD patients, which may be due to pathological changes and factors associated with AD, such as Aβ pathology, tau pathology, neuroinflammation, apolipoprotein E epsilon4 (ApoE ε4) allele, and advanced age. Focusing on BBB dysfunction in AD is of great significance for both the early detection of AD and the in-depth study of AD pathogenic mechanisms. Therefore, there is an urgent need to investigate the association between AD and BBB dysfunction and its underlying mechanisms. Here, we first summarized the evidence of BBB dysfunction in AD. Then, we analyzed the interrelationships and pathogenic mechanisms between AD and BBB dysfunction and the risk factors for AD, such as Aβ, tau, neuroinflammation, ApoE4 and aging. Finally, we discussed the current research status and potential future directions to reveal the mechanism by which the BBB contributes to AD pathology and develop BBB-targeted drug therapy for AD.

## 2. Molecular structure and functional characteristics of the BBB

The capillaries of the human brain are 650 km long, accounting for more than 85% of the total vascular length of the brain (Zlokovic, 2008; Pardridge, 2015). The BBB is a highly specialized brain endothelial cell (BEC) membrane (Zenaro et al., 2017). The BBB is mainly composed of BECs linked together by tight junction (TJ) proteins that are covered by a continuous basement membrane, pericytes and astrocyte terminal protrusions (Langen et al., 2019). The TJ complex is a unique molecular structure of the BBB and is composed of claudins, occludins, zonula occludens-1 (ZO-1), ZO-2, ZO-3, and adhesion molecules (Maiuolo et al., 2018). Pericytes share the basement membrane with BECs and regulate vascular permeability by releasing activating signals and controlling resting BEC differentiation (Langen et al., 2019). The protrusions of astrocytes extend into the capillaries with dilated ends and adhere as “end-feet” to the basement membrane of the vessels, which makes it more difficult for harmful substances to enter (Langen et al., 2019). Microglia are located outside the BBB and are able to sense microorganisms in the CNS and engulf and destroy those they encounter (Zenaro et al., 2017). Therefore, microglia are another line of immune defense against potential pathogens or toxins that cross the BBB. The majority of proteins and molecules do not pass directly through the BBB. Small molecules such as amino acids, glucose, vitamins and other trace elements needed by the brain need to be mediated through specific carriers, while biological macromolecules such as functional proteins and hormones require receptor-mediated transport to be able to pass through the BBB (Tiani et al., 2019). The BBB restricts the entry of blood products, circulating metals, pathogens and red blood cells into the brain, thereby stabilizing the internal environment of the brain tissue and ensuring that neurons and glial cells are able to work at their optimal level (Zlokovic, 2008; Sweeney et al., 2019). Future studies need to find effective targets for preventing or repairing neurovascular dysfunction, thereby slowing or halting the progression of AD.

## 3. Evidence of BBB dysfunction in AD

Due to vascular degeneration, altered molecular transport between the blood and brain, abnormal angiogenesis, cerebral underperfusion and inflammatory responses, BBB dysfunction may initiate and promote a “vicious cycle” of disease processes leading to progressive synaptic and neuronal dysfunction (Zlokovic, 2008). Numerous studies have shown evidence of the BBB-AD relationship in AD autopsy specimens, imaging data, cerebrospinal fluid, and animal models (Bowman et al., 2007; Zhao et al., 2015; Nelson et al., 2016; Wang et al., 2022). Here, we summarize the evidence for BBB dysfunction in AD (Tables 1, 2).

**Table 1:** Evidence of BBB dysfunction in AD patients.

Study subject	Methods	Main findings	References
Autopsy of AD patient	Immunohistochemistry and ELISA	Increased level of prothrombin in prefrontal cortex	Zipser et al. (2007)
Autopsy of AD patient	Immunohistochemistry	Diffused staining of fibrinogen and immunoglobulin in brain tissue	Ryu and McLarnon (2009)
Autopsy of AD patient	Immunocytochemistry, immunofluorescence	Cyclooxygenase-2 positive macrophages, and T and B-cell infiltration in brain perivascular spaces and neuropil	Fiala et al. (2002)
EOAD patients	DCE-MRI	Age-dependent dysfunction of BBB in hippocampus	Montagne et al. (2015)
EOAD patients	DCE-MRI	The permeability of BBB in total gray matter and cortex of brain increased significantly	van de Haar et al. (2016)
AD patients	3 T SWI	Approximately 46% of patients had microbleeds	Uetani et al. (2013)
MCI/AD patients	7Tesla MRI	Approximately 78% of patients had microbleeds	Brundel et al. (2012)
MCI/AD patients	^18^F-FDG PET	Significant changes in hippocampal formation and metabolism	Carlson et al. (2020)
AD patients	^11^C-verapamil PET	BBB P-gp activity in brain regions affected by AD was reduced	Deo et al. (2014)
MCI/AD patients	cerebrospinal fluid -Albumin	Increased or no change in Q-Alb	Skillback et al. (2017)
MCI/AD patients	cerebrospinal fluid -PDGFRβ	Increased level of PDGFRβ	Miners et al. (2019) and Nation et al. (2019)

EOAD, Early-onset AD; MCI, Mild cognitive impairment; ELISA, enzyme-linked immunosorbent assay; DCE, Dynamic contrast-enhanced; MRI, Magnetic resonance imaging; SWI, Susceptibility weighted imaging; ^18^FDG-PET, ^18^F-fluoro-2-deoxy-D-glucose positron emission tomography.

**Table 2:** Evidence of BBB dysfunction in animal models of AD.

Study subject	Methods	Main findings	References
APP/PS1 mice	Multiphoton microscope, histology, ethology, biology	Early progressive dysfunction of BBB and decrease of vascular density, capillary perfusion and cerebral blood flow	Cao et al. (2019)
5XFAD mice	Immunohistochemistry	Increased BBB permeability and decreased pericyte number	Giannoni et al. (2016)
P301L transgenic mice	Immunohistochemistry	Abnormal spiral shape of blood vessels with decreased diameter, and increased total vascular density of cortex	Bennett et al. (2018)
TgF344-AD rats	DCE-MRI	BBB permeability increased with age	Dickie et al. (2021)

FAD, Familial AD; DCE, Dynamic contrast-enhanced.

### 3.1. Evidence of BBB dysfunction in autopsy specimens

Evidence from postmortem brain tissue analysis demonstrated BBB dysfunction in AD patients (Zipser et al., 2007; Ryu and McLarnon, 2009). Common metrics include brain infiltration of circulating cells, leakage of blood-derived proteins (such as fibrinogen, thrombin and albumin), and structural changes in BBB components. Chen and Strickland (1997) highlights that laminin degradation catalyzed by circulating fibrinolytic enzymes disrupts neuronal extracellular matrix interactions and sensitizes hippocampal neurons to cell death. In addition, postmortem studies have demonstrated blood-derived protein accumulation, BBB-specific cell degeneration and vascular endothelial damage in the brain parenchyma of dementia patients (Wisniewski and Kozlowski, 1982; Slemmon et al., 1994). The postmortem brain results showed that peripheral macrophage infiltration was present in AD patients (Fiala et al., 2002). This finding may be due to increased glial cell activation in the brain and BBB dysfunction (Bettcher et al., 2021; Cisbani and Rivest, 2021). Although evidence from postmortem brain tissue of AD patients suggests that BBB dysfunction is a major player in AD pathology, it is difficult to exclude the possibility of BBB damage and changes in biochemical indicators due to lack of circulatory and internal environmental imbalance in the postmortem period by autopsy analysis. Moreover, it is difficult to obtain evidence of BBB damage and BBB dynamic changes in early AD from autopsy specimens.

### 3.2. Evidence of BBB dysfunction in neuroimaging data

Due to the rapid development of neuroimaging technology, AD diagnosis has also moved away from the previous stage of neuropathological biopsy/postdeath autopsy to realize the diagnosis of early BBB dysfunction (Bernal et al., 2021; Ohene et al., 2021). Dynamic contrast-enhanced magnetic resonance imaging (DCE-MRI) using a paramagnetic gadolinium-based contrast agent was able to detect BBB leakage in patients with early AD (Montagne et al., 2015). DCE-MRI results showed that compared with control subjects, early AD patients had significantly increased vascular leakage in gray matter and cortex, which correlated with cognitive decline (van de Haar et al., 2016). A 3 T susceptibility weighted imaging (SWI) study showed that approximately 46% of AD patients had microhemorrhages (Uetani et al., 2013), and the percentage of such microhemorrhages was even higher with 7Tesla MRI, rising to 78% (Brundel et al., 2012). In addition, the extent of coexisting vascular factors should be considered when interpreting BBB disruption. Cheng et al. (2023) noted that in AD patients, BBB injury is accompanied by severe cerebral small-vessel disease (CSVD), including microhemorrhages. The heterogeneity of concomitant vascular pathology in the AD brain may also contribute to the differences in these findings. These data provide experimental support for the role of BBB dysfunction in the pathophysiology of AD and suggest that more sensitive imaging techniques may help to further observe changes in the BBB in future studies.

In addition, abnormalities in BBB transporters are often observed in patients with mild cognitive impairment (MCI) and early AD (Szablewski, 2021). Brain uptake of the radiolabeled glucose analog ^18^F-fluorodeoxyglucose is dependent on glucose transporter proteins in the BBB endothelium (Kato et al., 2016). Recently, a study examined ^18^F-fluoro-2-deoxy-D-glucose positron emission tomography (^18^FDG-PET) in patients with AD and MCI and healthy individuals who served as controls. The results showed a significantly lower FDG standardized uptake value ratio (SUVr) in the dentate gyrus (DG) of AD/MCI patients and significantly lower volumes of cornu ammonis (CA1), DG and whole hippocampus in AD and MCI patients (Carlson et al., 2020). In addition, reduced transporter P-glycoprotein (P-gp) activity can be found by using ^11^C-verapamil PET imaging in AD patients (Deo et al., 2014). Detecting BBB transporter alterations by PET imaging provides new ideas and directions for exploring pathophysiological alterations in AD.

### 3.3. Evidence of BBB dysfunction in cerebrospinal fluid

Albumin (Alb) is a low molecular weight protein that is neither synthesized in the nervous system nor involved in its metabolism. Therefore, the ratio of clear protein in cerebrospinal fluid and blood, the albumin quotient (Q-Alb), is often used as a measure of BBB leakage (Musaeus et al., 2020). In several previous studies, Q-Alb was elevated in patients with AD and MCI (Skillback et al., 2017). However, a meta-analysis suggested that Q-Alb is not an appropriate biomarker for the diagnosis of AD (Olsson et al., 2016). Cerebrospinal fluid albumin levels may be influenced by protein hydrolytic cleavage and albumin uptake by brain macrophages, microglia, astrocytes, neurons, and oligodendrocytes (Braganza et al., 2012; LeVine, 2016). Therefore, it is advisable to use other measurements together with Q-Alb when assessing BBB permeability. Recently, Sweeney et al. (2020) developed a highly sensitive and reproducible standard assay protocol for soluble platelet-derived growth factor receptor β (PDGFRβ) immunoassay with a dynamic range of 100 to 26,000 pg/mL. Several studies have confirmed elevated levels of PDGFRβ in the cerebrospinal fluid of patients with AD and MCI (Miners et al., 2019; Nation et al., 2019). This suggests that PDGFRβ in cerebrospinal fluid could serve as a potential biomarker for BBB dysfunction in AD, which still needs to be confirmed by more studies (Sagare et al., 2015).

### 3.4. Evidence of BBB dysfunction in animal research

Pathological evidence of cerebrovascular and BBB dysfunction in AD transgenic mouse models has also been frequently reported (Montagne et al., 2017). Cao et al. (2019) found that early and progressive BBB dysfunction, vascular density, and cerebral blood flow decreased in APPswe/PS1Δe9 (APP/PS1) double transgenic AD model mice. In 5XFAD model mice that expressed mutations in the human amyloid precursor protein (APP) and Presenilin-1 (*PS1*) genes, increased BBB permeability and reduced PDGFRβ ^+^ pericyte numbers were observed (Giannoni et al., 2016). Another study found that P301L transgenic mice had an anomalous helical shape of cortical vessels, reduced vessel diameter, increased vessel density, and altered expression levels of angiogenesis-related genes [such as vascular endothelial growth factor A (*VEGFA*), serpin family E member 1 (*SERPINE1*) and urokinase-type plasminogen activator (*PLAU*)] (Bennett et al., 2018). Similar results were found in a 13-month-old transgenic rat model of AD with APPswe and PS1Δe9 mutations (TgF344-AD) (Dickie et al., 2021). In conclusion, evidence from animal studies suggests that BBB dysfunction is a major factor in AD pathology and that this may be an early event in AD.

The animal model obtained by simulating the aging process reproduces the pathological changes of AD more realistically. However, aging is only one of the risk factors for the development of AD, which is a pathological change that occurs on the basis of aging and is different from the normal physiological aging process, so the aging animal model cannot truly replace the AD model. Current research on AD has been conducted mainly in AD disease models, most of which are transgenic mouse models. There are a variety of transgenic mice that express genes related to AD, such as APP, PS1, tau, and ApoE. Mouse models carrying multiple mutations in APP showed Aβ deposition and cognitive deficits but no neurogenic fiber tangles or neuronal death (Li et al., 2023). Mouse models carrying a combination of mutations in APP and other genes [e.g., *PS1*, Presenilin-2 (*PS2*), and microtubule-associated protein tau (*MAPT*)] showed earlier and more aggressive pathological and behavioral changes than mouse models carrying only APP mutations (Li et al., 2023). However, the aging process in rodents does not fully reflect human pathology. One study used zebrafish as a model animal for functional studies and found that claudin-5-deficient zebrafish exhibit impaired BBB (Deshwar et al., 2023). Nonetheless, the utilization of zebrafish in AD-related BBB research is constrained due to its remarkable neuronal regenerative capabilities (Bhattarai et al., 2017). Compared to humans and mice, Drosophila is less relevant to humans and does not have the complex behavioral patterns of humans. Therefore, for more complex experiments, the use of more evolved non-human primates that are morphologically and genetically closest to humans may be a better choice for the future. It is worth noting that the relevance of physiological versus pathological brain aging cannot be fully captured by any current animal model. Since aging is the most important risk factor for late-onset AD, this is an important factor that cannot be fully captured in “advanced aging” models because they do not fully encapsulate the temporal and multifactorial aspects of human aging. Given that the neuropathological basis of AD remains unclear and that each model has its own advantages and limitations, comprehensive consideration should be given to selecting the most appropriate animal model for targeted studies.

## 4. BBB dysfunction accelerates AD

Many studies have demonstrated BBB damage in patients with AD, including vascular leakage, microhemorrhages, accumulation of perivascular blood-derived products, and dysfunction of the neurovascular units (NVUs) (Sweeney et al., 2018). Emerging studies have shown that many important pathological factors of AD, including Aβ, tau, neuroinflammation, ApoE4, and aging, are associated with BBB damage. We describe the relationship between BBB dysfunction and these AD pathological factors below.

### 4.1. BBB dysfunction correlates with AD pathology

#### 4.1.1. BBB dysfunction accelerates Aβ deposition

**Figure 1:**
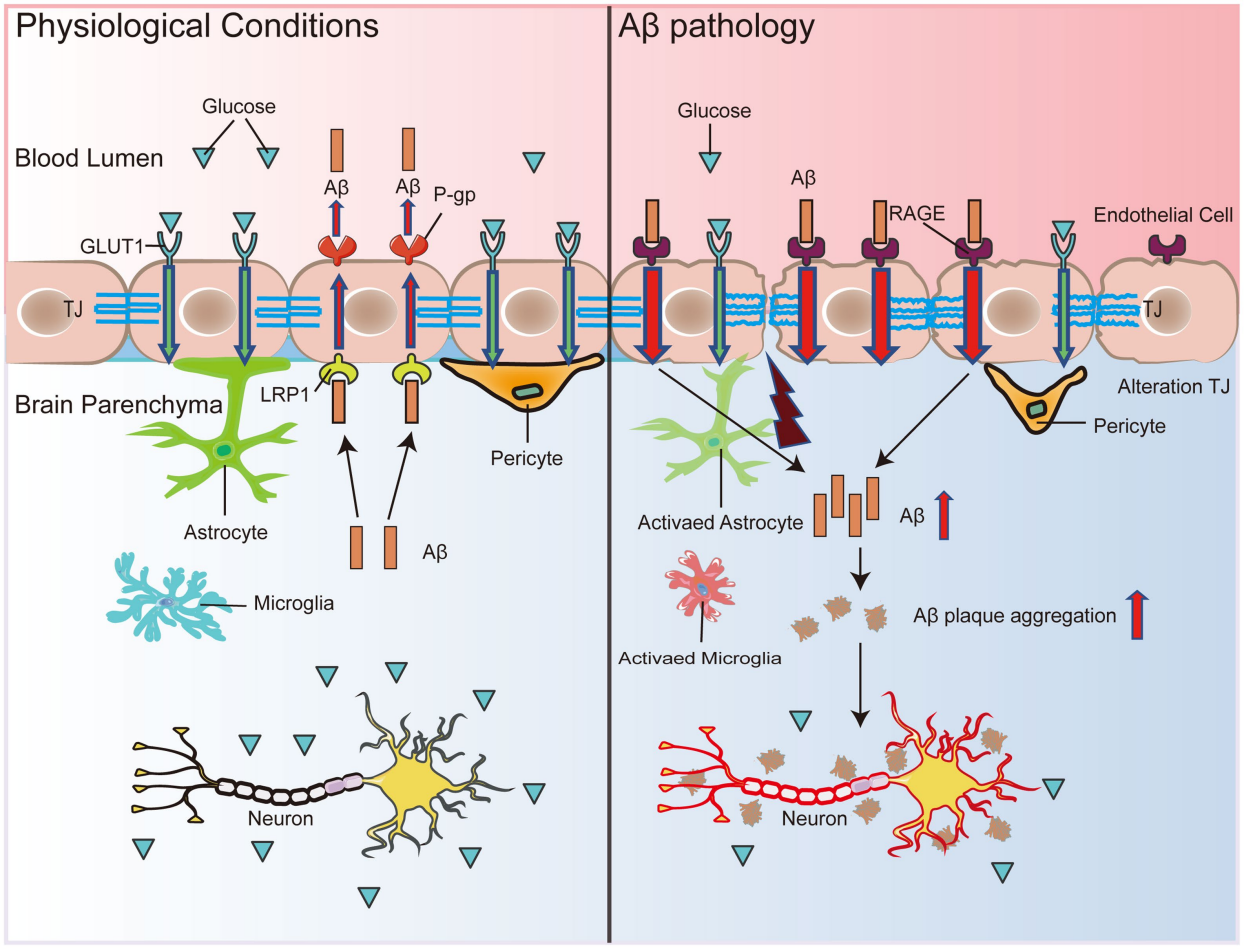
BBB dysfunction accelerates Aβ deposition. Under physiological conditions, Aβ binds to LRP1, and peripheral clearance of Aβ occurs with the assistance of P-gp, which transports Aβ out of the brain across the BBB. Simultaneously, RAGE expressed by endothelial cells can bind to peripheral free Aβ to reuptake it into the brain. When LRP1 expression decreases and RAGE expression rises in the BBB, peripheral clearance of Aβ is blocked, and re-entry into the brain increases, which leads to Aβ accumulation in the brain. In addition, decreased GLUT1 expression in endothelial cells leads to BBB disruption and transcriptional repression of LRP1, which accelerates Aβ pathology and leads to secondary neurodegenerative changes, neuronal loss, and brain atrophy. Thus, the malignant accumulation of Aβ in the brain may become a destructive feedback loop that may eventually induce neuronal damage in the brain, leading to impaired cognitive and memory functions.

Aβ production is a process in which APP is sequentially cleaved first by β-secretase and then by γ-secretase (Atwal et al., 2011). In animal models of AD, 70–85% of Aβ in the brain is mainly cleared across the BBB, with only a very small fraction cleared through intercellular fluid (Shibata et al., 2000). Aβ deposition is highly heterogeneous and contains multiple fragments, mainly from the catabolism of Aβ_40_/Aβ_42_, which exhibit different aggregation properties (Hartz et al., 2012). Aβ peptides that mainly adopt the protofibrillar conformation, such as Aβ_42_ and Aβ_4-42_, have the greatest effect on BBB permeability, while peptides that mostly remain as monomers in the form of Aβ_1-16_ and Aβ_1-34_ or form low molecular weight oligomers such as Aβ_40_ and Aβ_4-34_ have the greatest effect on EC monolayer transendothelial electrical resistance (Hartz et al., 2012). Numerous studies have shown that lipoprotein receptor-related protein-1 (LRP1) and receptor for advanced glycation end products (RAGE) in the BBB are essential for regulating Aβ homeostasis in the brain (Moir and Tanzi, 2005; Sharma et al., 2012). In a physiological state, Aβ binds to LRP1, and peripheral clearance of Aβ occurs with the assistance of the adenosine triphosphate (ATP)-binding cassette transporter protein P-gp, which transports Aβ out of the brain across the BBB (Storck et al., 2018; Erdo and Krajcsi, 2019; Figure 1). Simultaneously, RAGE expressed by BEC can bind to peripheral free Aβ to reuptake it into the brain (Cai et al., 2016; Figure 1). When LRP1 is reduced or RAGE is increased on the BBB, peripheral clearance of Aβ is blocked, and re-entry of Aβ into the brain is increased, accelerating Aβ accumulation in AD (Shang et al., 2019; Figure 1). In addition, BBB dysfunction may activate β-secretase and γ-secretase cleavage of APP, ultimately leading to Aβ overproduction (Atwal et al., 2011; Zhang et al., 2013). In the AD state, Aβ production far exceeds the clearance capacity of the BBB, exacerbating Aβ accumulation in the brain (Parodi-Rullan et al., 2021). Aβ aggregation is observed in the vessel walls of most patients with AD, a phenomenon or disease called cerebral amyloid angiopathy (CAA) (DeTure and Dickson, 2019). Moreover, the number of BBB TJ proteins was reduced by 30% to 40%, and glial cell aggregation and activation and fibrinogen leakage into the brain parenchyma were presented (Carrano et al., 2012). Aβ oligomers were found to act directly on BECs to inhibit their wingless and int-1 (Wnt)/beta-catenin (β-catenin) signaling, thereby disrupting BBB function (Wang et al., 2022).

Furthermore, several studies performed in MCI patients and early AD patients have shown that decreased glucose transporter 1 (GLUT1) expression suppresses glucose transport through the BBB prior to brain atrophy, neurodegenerative manifestations, or conversion to AD, leading to impaired local glucose uptake, which in turn causes inadequate neuronal energy sources (Simpson et al., 1994; Winkler et al., 2015). Reduced expression of GLUT1 in BEC triggers the damage of the BBB and transcriptional repression of LRP1, which accelerates Aβ pathology and ultimately causes glial cell inflammatory responses, neuronal damage, and brain atrophy (Figure 1). Aβ deposits impair the ability of the BBB to transport nutrients, remove metabolic waste, and prevent the invasion of harmful substances, leading to worsening vascular pathology in the AD brain, further neurodegeneration, and a vicious cycle leading to progressive cognitive decline (Figure 1).

#### 4.1.2. BBB dysfunction expedites tau formation and dispersion

**Figure 2:**
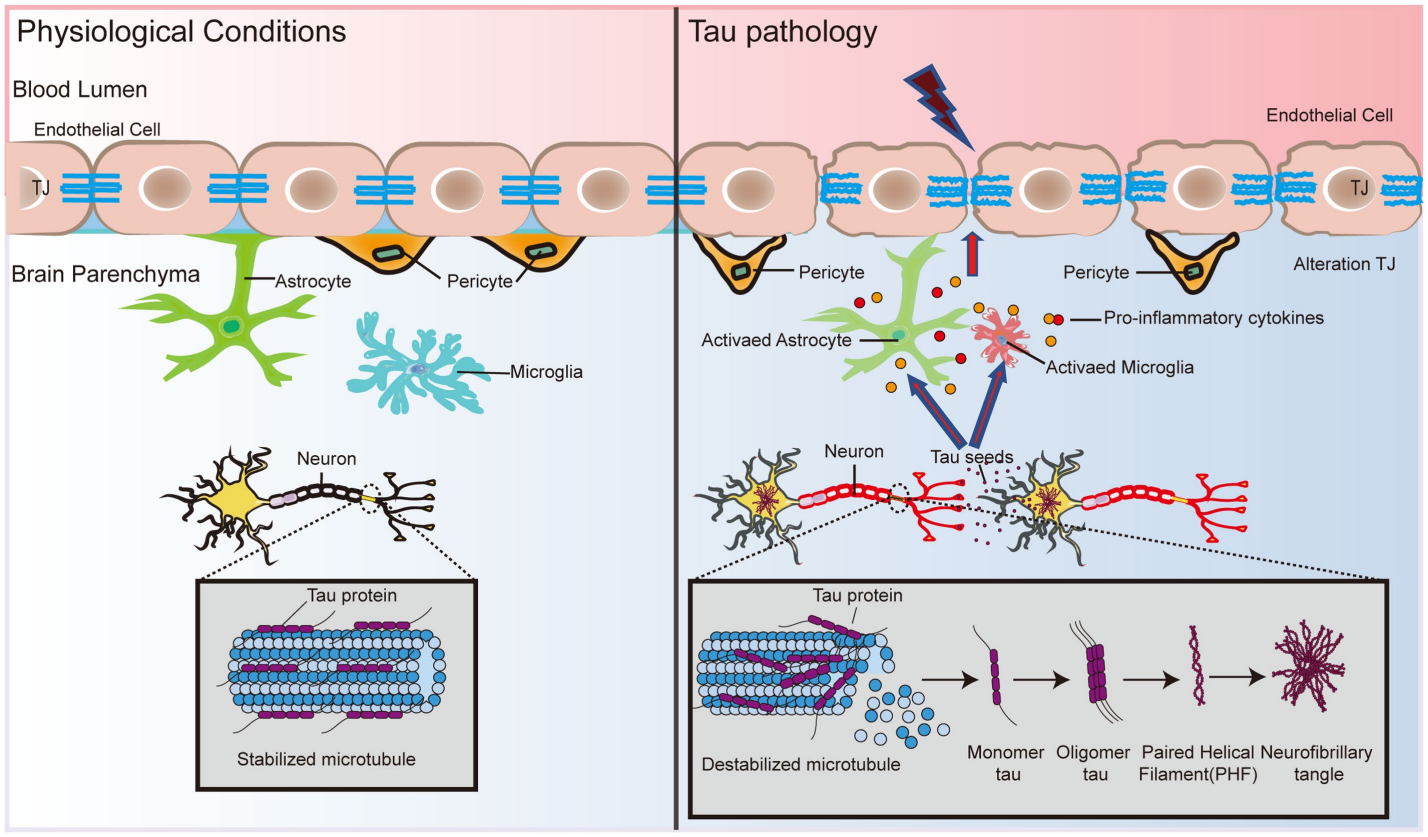
BBB dysfunction expedites tau formation and dispersal. Tau protein status determines the binding and stability of microtubule protein aggregates in neuronal cells. Hyperphosphorylation causes tau protein to lose its ability to bind microtubules, which triggers the formation of PHF and subsequently leads to the formation of NFTs. In addition, tau protein interacts with glial cells and leads to the release of proinflammatory cytokines, causing the dysfunction of TJ proteins, atrophy of pericytes, and disruption of basement membranes, which in turn exacerbates damage to the BBB. Eventually, the pathological effects of tau in the brain may spread to endothelial cells and pericytes, inducing and accelerating BBB dysfunction, which in turn leads to neuronal activity dysfunction.

Neurofibrillary tangles (NFTs) are another major pathological feature in AD and are formed by abnormal aggregation of neuroprogenitor fibers, the main component of which is highly phosphorylated tau (P-tau). Overphosphorylation inhibits tau binding to microtubules, resulting in the formation of paired helical filaments (PHFs), which then form NFTs in AD (Crespo-Biel et al., 2012; Figure 2). These alterations trigger instability of the cytoskeleton (Kolarova et al., 2012), leading to impaired transport of substances, degeneration of nerve fibers and ultimately cell death (Liu et al., 2007; Figure 2).

Although tau has not been studied as extensively as Aβ in AD pathology, the association between tau and the BBB will be further explored as the importance of tau in AD becomes increasingly recognized. A recent study showed that cultured primary rat BECs exposed to oligomeric tau changed the endothelial properties of the BBB and promoted the migration of blood to brain cells (Majerova et al., 2019). Using a tau disease transgenic rat model (SHR-72), Majerova et al. (2019) found numerous undulating protrusions on the luminal surfaces of BECs, markedly inhomogeneous basement membranes, and twisted or distorted TJs. Interestingly, direct exposure of BEC to aggregated tau protein alone did not elicit any significant response, whereas migration of peripheral blood monocyte-derived macrophages (PB-MoM) increased 24 h after addition of aggregated tau in the presence of glial cells (Majerova et al., 2019). This finding indicates that glial cells drive neuroinflammation in tau lesions and may accelerate BBB damage (Figure 2). Glial cells activate and release proinflammatory cytokines, including interleukin-1beta (IL-1β), tumor necrosis factor-alpha (TNF-α), and interleukin-6 (IL-6), which alter endothelial properties (Zilka et al., 2009; Lee et al., 2010; Figure 2). The progressive increase in immunoreactive cells with incremental NFT loading suggests that activated glial cells promote the immune response to tau neurogenic fiber pathology (Zilka et al., 2009). This finding suggests that the crosswalk between oligomeric tau proteins and glial cells causes neuroinflammation, and disruption of TJ proteins disrupts pericytes and basement membranes, thereby exacerbating BBB damage (Figure 2). In addition, BBB dysfunction impedes the clearance of oligomeric tau proteins (Tarasoff-Conway et al., 2015). Excess tau is released outside the cell and internalized by the surrounding neurons and cells, which promotes the diffusion of tau throughout the brain (Bolos et al., 2016). Tau lesions induce and accelerate BBB damage and circulating immune cell permeability, eventually spreading throughout the cortex, leading to neurological failure, neurodegeneration, and cognitive decline (Figure 2).

#### 4.1.3. BBB: an important bridge between neuroinflammation and systemic inflammation

**Figure 3:**
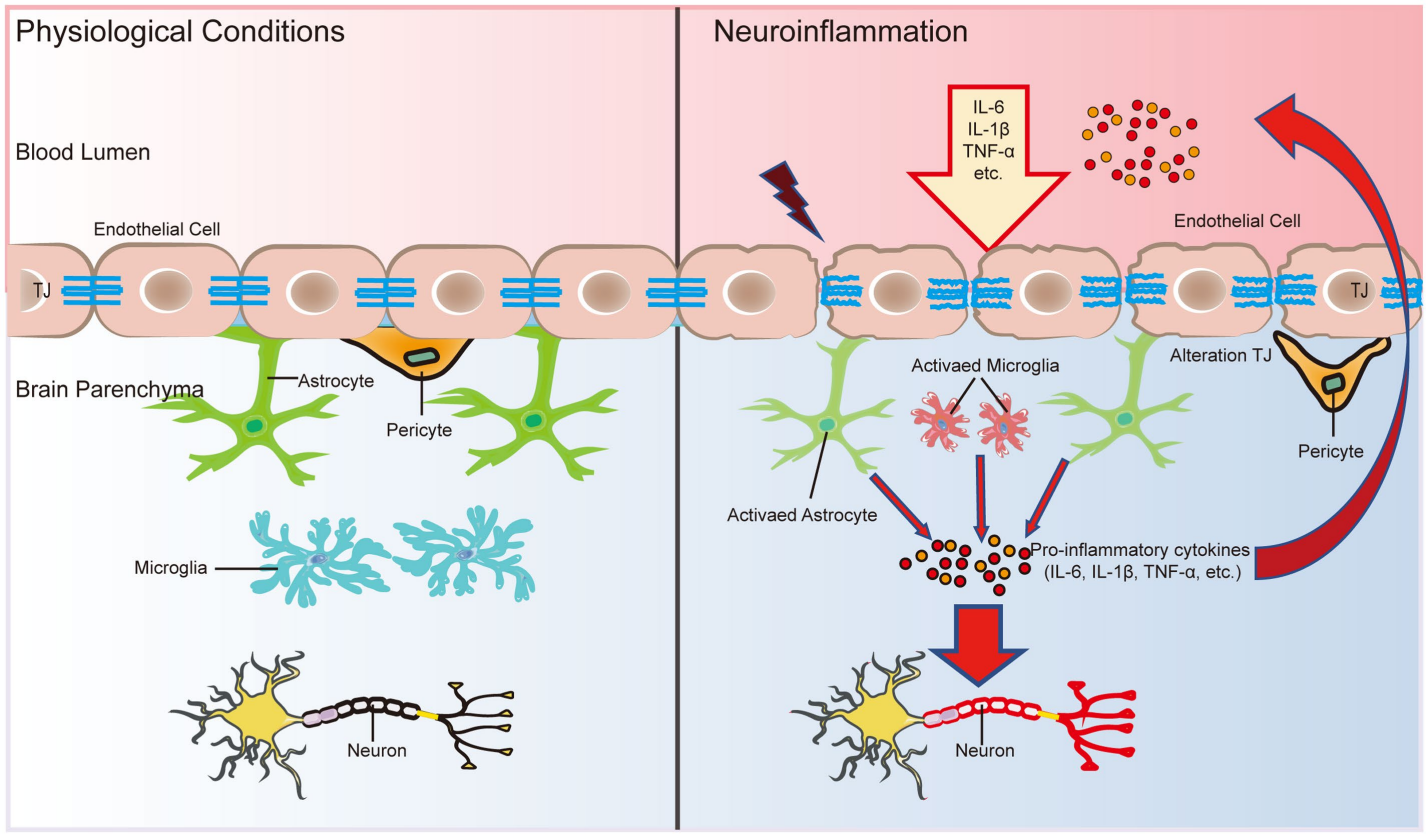
The BBB is an important bridge between neuroinflammation and systemic inflammation. The main factors that disrupt the BBB and induce neurodegeneration are three inflammatory cytokines: IL-1β, TNF-α, and IL-6. In addition, inflammatory cytokines in the CNS can activate glial cells and alter their function, leading to microglial activation and further release of proinflammatory cytokines to breakdown TJ proteins, further disrupting the functional and structural integrity of the BBB. In turn, the early and progressive perivascular activation of glial cell aggregation triggered by BBB dysfunction contributes to neuronal damage in neuroinflammatory diseases.

The chronic inflammatory microenvironment in the AD brain shows high levels of CNS glial cells, including microglia and astrocytes, that produce proinflammatory cytokines after being activated through pattern recognition receptors (PRRs) or ATP (Chen et al., 2021). Inflammatory cytokines trigger the upregulation of matrix metalloprotease 9 (MMP9) in BECs, which attacks components of the endothelial basal layer and TJs (Yang et al., 2016). The main inflammatory cytokines that damage the BBB and induce neurodegeneration are IL-1β, TNF-α, and IL-6 (Yang et al., 2022; Figure 3). IL-1β increases BBB leakage and exacerbates CNS degeneration by activin receptor-like kinase (ALK)-small mothers against decapentaplegic (SMAD) pathway (Sun et al., 2022). Aslam et al. (2012) found that TNF-α reduced claudin-5 expression levels by activating the nuclear transcription factor-kappa B (NF-κB) signaling pathway, thereby disrupting TJ structure. IL-6 levels were increased by the activation of astrocytes induced by TNF-α and IL-1β through the NF-κB signaling pathway, while IL-6 was negatively regulated by the Wnt/β-catenin pathway (Edara et al., 2020). In addition, activation of C3a receptors in BEC resulted in increased BBB permeability due to increased intracellular calcium ion (Ca^2+^) levels disrupting vascular endothelial calmodulin-based adhesion junctions (Propson et al., 2021). The dysfunction of the BBB, in turn, enhances the inflammatory response of the brain.

During inflammation, neutrophils and T lymphocytes outside the CNS are recruited into the CNS via cytosis or the paracellular pathway to secrete proinflammatory cytokines and chemokines, which further disrupt the integrity of the BBB and exacerbate brain tissue damage (Carrano et al., 2012). Cytokines secreted by immune cells disrupt TJs at a late stage to open the paracellular pathway, which allows more immune cells to cross the BBB, especially T-helper 1 (Th1) cells and T-helper 17 (Th17) cells (Liebner et al., 2018). There is a high level of neutrophil infiltration in the AD hippocampus, which leads to the production of the neurotoxic cytokine interleukin-17 (IL-17) and neutrophil extracellular traps (Zenaro et al., 2015). An intense inflammatory reaction may produce autotoxicity in neurons, thus exacerbating the progression of AD and other nervous system diseases (McGeer and McGeer, 1995; Figure 3). In addition, the entry of proinflammatory cytokines into the CNS can trigger the activation and proliferation of microglia and further damage the integrity of the BBB (John et al., 2003; Figure 3). Microglial activation and proliferation decompose TJ proteins by releasing proinflammatory cytokines and promoting oxidative stress (Shigemoto-Mogami et al., 2018; Figure 3). Damage to the BBB recruits more immune cells and cytokines into the brain parenchyma, which in turn induces neurodegeneration. Infiltrating immune cells, microglia and astrocytes may activate each other, driving chronic inflammation and preventing BBB repair, accompanied by pericyte damage and neuronal loss, thus further contributing to cognitive decline (Figure 3).

### 4.2. ApoE4: increased susceptibility to BBB dysfunction

**Figure 4:**
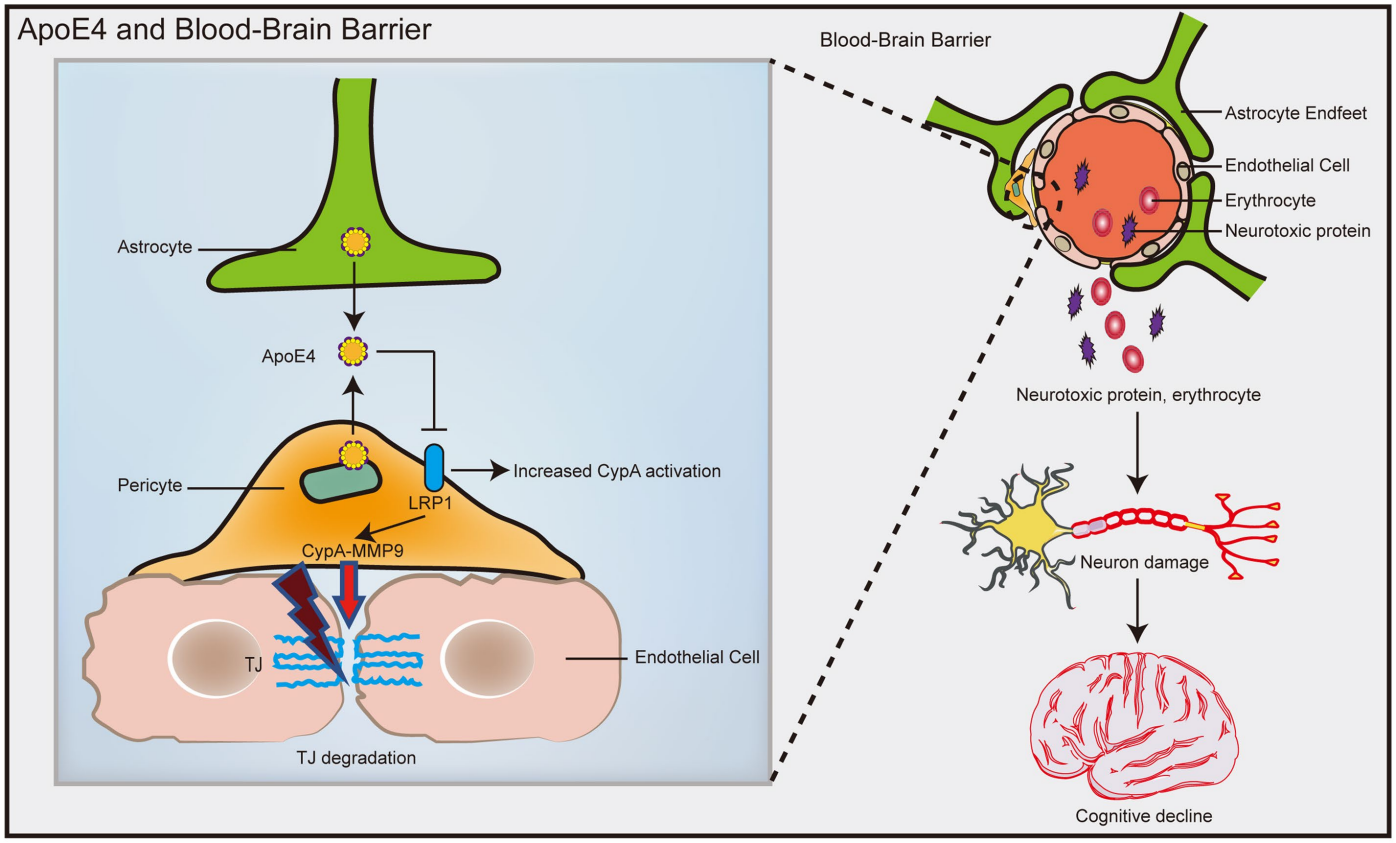
ApoE4 contributes to increased susceptibility to BBB dysfunction. ApoE4 derived from astrocytes and pericytes leads to decreased LRP1 expression on pericytes, resulting in activation of the proinflammatory signal CypA/MMP9, which in turn disrupts the TJ between endothelial cells. Disruption of the BBB leads to neuronal uptake of multiple blood-derived neurotoxic proteins, as well as reduced microvascular and cerebral blood flow, which in turn causes secondary neuronal damage and cognitive decline.

In humans, ApoE has three isoforms: ApoE2, ApoE3, and ApoE4. ApoE4 is the most common risk factor for sporadic AD (Verghese et al., 2011), and it was recently identified as the most overlapping gene between AD and vascular pathology (Lin et al., 2019). ApoE4 transgenic model mice showed an increase in BBB vulnerability (Nishitsuji et al., 2011; Bell et al., 2012), while the BBB integrity of mice carrying ApoE2 and ApoE3 was not affected (Bell et al., 2012). Compared with ApoE3 carriers, AD patients who are ApoE4 carriers have significantly increased pericellular degeneration and BBB dysfunction (Halliday et al., 2016; Riphagen et al., 2020). Relative to 12-month-old ApoE2 and ApoE3 mice, the cerebral vascularization of ApoE4 mice decreased structurally, and the basement membrane became thinner, indicating that the blood vessels were atrophied (Alata et al., 2015). A recent population-based study found that in elderly individuals with normal cognition, ApoE4 gene carriers showed significant BBB leakage, while ApoE3 carriers did not exhibit BBB leakage until the mild cognitive dysfunction stage (Montagne et al., 2020). In late AD patients, plasma proteins such as prothrombin can be found in microvascular walls and peripheral nerve membranes, and BBB leakage may be more common in patients with at least one ApoE ε4 allele (Zipser et al., 2007). Human ApoE4 induces cholesterol/lipid homeostasis dysregulation, increased inflammatory signaling, and decreased β-amyloid uptake in astrocytes generated from pluripotent stem cells (de Leeuw et al., 2022). Jackson et al. (2022) found that ApoE4 derived from astrocytes increased the permeability of the BBB by specifically upregulating the expression of MMP9 and destroying TJs and astrocyte terminal feet (Figure 4). In addition, Halliday et al. (2016) found a significantly faster rate of intracerebral pericyte loss in ApoE4 carriers than in ApoE3 carriers in autopsies of AD patients. Subsequent studies in ApoE4 transgenic mice showed that reduced levels of LRP1 in mouse brain pericytes led to activation of the proinflammatory signaling pathway cyclophilin A (CypA)/MMP9, which in turn damaged the TJs between BECs (Halliday et al., 2016; Figure 4). The dysfunction of the BBB mediated by the CypA-MMP9 pathway leads to the uptake of neurotoxic proteins from various blood sources by neurons, as well as a reduction in microvascular and cerebral blood flow, thus causing secondary neuronal damage and cognitive decline (Figure 4).

### 4.3. Aging: a primary risk factor for BBB

Aging is an important factor that accelerates BBB dysfunction (Tibbling et al., 1977). Tables 3, 4 summarize the results of studies on age and BBB dysfunction. In the aging process, the loss of TJs, a decrease in astrocyte end foot coverage and the hardening of blood vessels were observed in the BBB (Marques et al., 2013). Compared with 3-month-old young control mice, 24-month-old C57BL/6 J mice exhibited significantly increased immunoglobulin G (IgG) exosmosis and significantly increased expression of glial fibrillary acidic protein (GFAP), 78-kDa glucose-regulated protein (GRP78) and cyclooxygenase-2 (COX-2) in the cerebral cortex and hippocampus. Additionally, the expression of the TJ protein occludin and, to a lesser extent, ZO-1 in the BBB of 24-month-old C57BL/6 J mice was significantly reduced compared with that in young mice (Elahy et al., 2015). In aged mice, receptor-mediated endocytosis transport was significantly reduced, while nonreceptor-mediated (nonspecific) endocytosis transport was increased, resulting in nonspecific entry of plasma proteins into the brain (Munji and Daneman, 2020). Pelegri et al. (2007) noted that endogenous IgG extravasation levels were significantly higher in brain microvessels in the hippocampus of 12-month-old senescence-accelerated mouse prone 8 (SAMP8) mice than in control nonsenescent (susceptible to accelerated senescence, SAMR1) mice. Ueno et al. (1993) reported that the rates of radioactive serum albumin transfer in the parietal cortex and hippocampus of 22-month-old SAMR1 mice and the hippocampus of 13-month-old SAMP8 mice were higher than those of young mice of the corresponding genetic background. BBB disruption in the hippocampus of senescent SAMP8 mice may underlie learning and memory deficits. The cortices of young (2–3 months) and old (18–22 months) C57BL/6 J mice were analyzed using single-nucleus RNA-sequencing (snRNA-seq) and found to be enriched for differentially expressed genes in TJs, the TGF-β signaling pathway and the inflammatory signaling pathway between BBB-associated and non-BBB-associated clusters (Zhan et al., 2023). Among them, connexin 43 (Cx43) downregulation in cadherin-5 (Cdh5)^+^ cell is one of the most significant changes in the natural aging process of the BBB, and Cx43 deficiency leads to cognitive dysfunction during aging (Zhan et al., 2023).

**Table 3:** The manifestation of the aging BBB in humans.

Study subjects	Age	Method	Main findings	References
NCI and ECI individuals	45 years of age	DCE-MRI	- BBB permeability increases with age in the hippocampus and hippocampal subregions such as CA1 and dentate gyrus in normal elderly individuals. - The integrity of the BBB is further disrupted during the stage of MCI.	Nation et al. (2019)
NCI individuals and MCI patients	NCI: (23–47), (55–91) years old, MCI: (55–85) years old	DCE-MRI	Increased BBB permeability in the hippocampus in older adults, which is more severe in individuals with MCI	Montagne et al. (2015)
Autopsy of AD patient	-	- Immunofluorescence staining - Fluorescence analyses	- Significant extravascular accumulation of brain parenchymal fibrinogen - Significantly reduced expression of claudin-1 and GLUT1	Wang et al. (2022)

NCI, Noncognitive impairment; ECI, Early cognitive impairment; MCI, Mild cognitive impairment.

**Table 4:** The manifestation of the aging BBB in animal models.

Study subjects	Age	Method	Main findings	References
C57BL/6 J mice	3-month-old 24-month-old	- Three-dimensional semiquantitative immunomicroscopy, - Flow cytometry analysis	- Circulating IgG infiltration into brain parenchyma in aged mice - GFAP, GRP78 and COX-2 expression was significantly increased in the cerebral cortex and hippocampus of aged mice. - The expression of TJ protein, Ocludin-1 and ZO-1 was attenuated in aged mice.	Elahy et al. (2015)
Young adult mice Aged mice	-	Labeled endogenous mouse plasma protein	Significantly reduced receptor-mediated cytokinesis transport and increased nonreceptor-mediated cytokinesis transport in aged mice	Munji and Daneman (2020)
SAMR1 mice SAMP8 mice	3-month-old, 7-month-old, 12-month-old	- Immunofluorescence staining - Fluorescence analyses	The level of IgG extravasation was significantly elevated in the hippocampus of 12-month-old SAMP8 mice.	Pelegri et al. (2007)
SAMR1 mice SAMP8 mice	3-, 13-, and 22-month-old (SAMR1), 3-, 7-, and 13-month-old (SAMP8)	Dual Isotope Technology	The rates of radioactive serum albumin transfer in the parietal cortex and hippocampus of 22-month-old SAMR1 mice and the hippocampus and cerebellum of 13-month-old SAMP8 mice were high.	Ueno et al. (1993)
C57BL/6J mice	2–3 months 18–22 months	snRNA-seq	Cx43 is significantly downregulated in old mice	Zhan et al. (2023)
C57BL/6J mice APP/PS1 mice	2-, 4-, and 9-month-old	- Immunofluorescence staining - Fluorescence analyses	- Significant extravascular accumulation of fibrinogen in the brain parenchyma of 9-month-old APP/PS1 mice - The expression of claudin-1 and GLUT1 in 9-month-old APP/PS1 mice was significantly reduced	Wang et al. (2022)

snRNA-seq, Single-nucleus RNA-sequencing.

Aging is considered an inevitable and irreversible life process. BBB leakage occurs in normal aging as well as in AD. BBB dysfunction is also strongly associated with cognitive impairment and AD in most older adults (Farrall and Wardlaw, 2009). Recently, increases in BBB permeability of the hippocampus and other subregions during normal aging have been preliminarily confirmed by using improved DCE-MRI technology (Nation et al., 2019). During the stage of MCI and AD, BBB integrity is further disrupted, and its increased permeability is significantly correlated with cerebrospinal fluid soluble PDGFRβ levels and precedes cerebrospinal fluid Aβ and tau protein abnormalities (Nation et al., 2019). Human high-resolution MRI shows that the loss of BBB integrity in the hippocampus gradually increases with age, which is more serious in individuals with MCI (Montagne et al., 2015). Dysfunction of the BBB in AD is manifested by the accumulation of blood-borne fibrinogen in the hippocampus and perivascular areas of the cortex and is accompanied by reductions in TJ, claudin-5, and GLUT1 in BECs (Wang et al., 2022). In the APP/PS1 mouse, BBB dysfunction began at 4 months, became severe at 9 months, and progressively worsened with age (Wang et al., 2022). With healthy aging, the BBB undergoes many changes that may be adaptive but may also be reactive to age-related diseases. Aging makes the BBB of AD more susceptible to inflammatory cytokines, and BBB injury tends to invite more immune cells and cytokines into the brain parenchyma, thus inducing neuroinflammation. Therefore, AD should be detected and diagnosed early, and appropriate interventions should be taken at an early stage to prevent and slow the development of dementia.

## 5. Therapeutic potential

### 5.1. Repair and closure of dysfunctional BBB

When the BBB malfunctions, the leaking proteins and cells alter the environment of the nerve cells, leading to nerve cell death and disease progression. Since cerebrovascular factors are associated with AD disease, repairing cerebrovascular injury may slow the disease process of cognitive impairment (Kang et al., 2010; Paul et al., 2012). Angiotensin receptor blockers (ARBs) restore the structural integrity of the BBB, increase the expression of claudin-5 and ZO-1 in aged rats, and improve cognitive function (Rodriguez-Ortiz et al., 2023). In contrast, repair of the BBB with activated protein C (APC) and its cytoprotective analogs not only protects BBB integrity but also slows the progression of neurological diseases (Winkler et al., 2014; Griffin et al., 2015). APC enhances endothelial membrane integrity by activating renin angiotensin system (Ras)-related C3 botulinum toxin substrate 1 (Rac1)-dependent cytoskeletal stabilization and downregulating MMP9 in the BBB, thereby protecting the glial environment from systemic effects (Zlokovic, 2011). A recent study in a 5XFAD mouse model showed that treatment with 3K3A-APC, a cell signaling analog of the endogenous hemoproteinase APC, improved BBB integrity and 40–50% parenchymal and cerebrovascular Aβ deposition formation (Lazic et al., 2019). Inhibition of the pro-inflammatory CypA-MMP9 pathway of BBB degradation in pericytes using the CypA inhibitor Debio-025 improved BBB integrity and prevented cognitive decline in E4FAD mice (produced by crossing 5XFAD with ApoE4*^+/+^*-targeted replacement mice) (Montagne et al., 2021). The study of the gut microbiome is rapidly expanding with the recent development of high-throughput bioassays, allowing us to better understand the composition and function of such a complex ecosystem. Furthermore, through DCE-MRI technology, it was found that transplantation of induced pluripotent stem cell (iPSC) pericytes into the hippocampus of *Pdgfrb^F7/F7^* mice with peripheral cell defects can restore BBB permeability and increase neuronal count and neurite density in the hippocampus (Allison Bosworth et al., 2023). Thus, replacing dysfunctional pericytes with cell therapy has the potential to restore impaired BBB and brain function (Faal et al., 2019).

In addition, the human gut harbors approximately 100 trillion microorganisms, which are implicated in the integrity of the intestinal epithelial barrier, intestinal metabolism, and the maintenance of immune homeostasis (Valdes et al., 2018). Gut microbes and metabolites are regulators of BBB integrity and brain health (Parker et al., 2020). Compared to conventionally reared mice, germ-free mice have decreased expression of TJ-related proteins such as occludin and claudin-5, resulting in increased BBB permeability (Braniste et al., 2014). In germ-free mice colonized with *Clostridium tyrobutyricum* or *Bacteroides thetaiotaomicron* producing short-chain fatty acids, expression of the TJ proteins occludin and claudin-5 was enhanced and BBB permeability decreased (Braniste et al., 2014). Feeding Bifidobacterium improves intestinal dysbiosis, decreases fecal and blood levels of lipopolysaccharide and inflammatory factors, and increases the expression of TJ-related proteins in 5XFAD mice, which reduces Aβ load in the brain and improves cognitive function (Lee et al., 2019). In a randomized, double-blind, controlled clinical trial, after continuous administration of probiotic milk (containing Lactobacillus and Bifidobacterium) to AD patients for a total of 12 weeks, the patients experienced a significant decrease in the levels of inflammatory factors in their bodies and a reduction in BBB leakage (Akbari et al., 2016). In addition, fecal microbiota transplantation (FMT) is a therapeutic modality to transplant the intestinal microflora of a healthy donor into the intestinal tract of a patient, and the intestinal flora of patients who receive FMT can be comprehensively regulated so that part of the intestinal flora homeostasis can be reestablished or restored. Animal model studies have found that transplantation of feces from wild-type mice into AD mice (5XFAD, APP/PS1 mice) for 4 consecutive weeks can reduce Aβ deposition in the brains of AD mice, attenuate glial cell hyperactivation and the level of neuroinflammation secondary to it, and attenuate BBB permeability, thus improving the cognitive function of the mice (Sun et al., 2019; Kim et al., 2021). Thus, probiotic supplementation and FMT, among other means, may influence the composition of the intestinal microbiota and the production of its metabolites, which, by mediating structural and functional alterations of the BBB, may in turn affect AD neuropathology and cognitive function.

### 5.2. Eliminating the consequences of BBB dysfunction

When the BBB opens, plasma proteins enter the glial space and become neurotoxic. Therefore, neutralizing toxic accumulation may be a valuable therapeutic approach for the treatment of neurodegenerative diseases associated with BBB pathology. Indeed, depletion of accumulated fibrinogen in the brain with ancrod (a fibrinogen-removing agent) or by genetic manipulation reduces neuroinflammation and vascular lesions in APP mice (Paul et al., 2007). On the other hand, brain accumulation of BBB damage causing red blood cell extravasation and free neurotoxic iron causing oxidative stress could be successfully controlled by iron chelators and antioxidant treatment (Winkler et al., 2014). A recent study showed that deletion of brain endothelial LRP1 leads to loss of BBB integrity, neuronal loss, and cognitive deficits in *Lrp1^lox/lox^* mice (an endothelial-specific *Lrp1* knockout mouse) and that these deficits can be reversed by endothelial-specific LRP1 gene therapy (Nikolakopoulou et al., 2021). Compared to the vector, artesunate-treated 5XFAD mice showed a 2-fold increase in brain capillary phosphatidylinositol-binding clathrin assembly protein (PICALM) levels and a 34–51% reduction in Aβ_42_ and Aβ_40_ levels, Aβ and thioflavin S load, and vascular Aβ load in the cortex and hippocampus (Kisler et al., 2023). Artesunate also increased circulating Aβ_42_ and Aβ_40_ levels by 2-fold, confirming accelerated clearance of Aβ from the brain to the blood (Kisler et al., 2023). In addition, a high-affinity RAGE-specific inhibitor (FPS-ZM1), which blocked Aβ binding to the V domain of RAGE, reduced brain levels of Aβ_40_ and Aβ_42_ and normalized cognitive performance and cerebral blood flow responses in aged *APP^sw/0^* mice (Deane et al., 2012).

### 5.3. Increased efficiency of drug delivery to targeted brain tissue

While the BBB provides for the dynamic and complex physiological environment of the CNS due to its properties, it also serves as a critical barrier to intracerebral drug delivery. This results in almost all large molecule drugs and over 98% of small molecule drugs failing to cross the BBB (Zhang et al., 2016). The main problem with therapeutic compounds is that they lose bioavailability, solubility, stability, or efficacy before reaching the target site. Therefore, researchers have proposed treatment measures to increase the efficiency of drug delivery to brain tissue.

Currently, one main approach is to encapsulate or load the drug on a suitable carrier to pass it through the BBB. Drug-loaded nanoparticles (NPs) enhance the permeability of the therapeutic compound through the BBB, thus reaching the corresponding target in the brain without losing its properties and thus treating the lesion more effectively and precisely (Zhong et al., 2023). At present, strategies to increase the brain penetration and potency of neurotherapeutic agents using existing carrier-mediated cytokines (CMT) (Erickson and Banks, 2013) and receptor-mediated cytokines (RMT) (Jones and Shusta, 2007; Niewoehner et al., 2014; Yu et al., 2014; Bray, 2015) of the BBB system have been explored. Zhou et al. (2020) found that Gal-NP@siRNA (a glycosylated “triple-interaction” stabilized polymeric siRNA nanomedicine) had good blood stability and could effectively penetrate the BBB through glucose-controlled GLUT1-mediated transport and improve cognitive dysfunction in AD mice without significant side effects. An increasing number of researchers are working on new nanotechnology strategies to improve drug delivery in the CNS. Zhang et al. (2023) used the natural Ca^2+^ antagonist magnesium ion (Mg^2+^) and siRNA targeting the core regulatory factor cyclophilin D (CypD) of the mitochondrial permeability transition pore (mPTP) to be encapsulated into the nanobrake to achieve targeted delivery of brain mitochondrial dysfunction cells across the BBB. Similarly, the covalent organic framework (SC@COF-T5) functionalized with amyloidogenic peptide fragment KLVFF (T5) and loaded with superoxide dismutase and catalase possessed excellent ROS scavenging activity, and the modification of the targeting peptide T5 endowed the nanoparticles with the ability to cross the BBB and bind to brain Aβ (Ren et al., 2023). In contrast, genetically engineered neural stem cell membrane-encapsulated traceable nanopreparations (RVG-NV-NPs) were able to monitor and evaluate *in vivo* their blood circulation, BBB crossing, and neuronal targeting processes without affecting the targeting properties of the nanopreparations, and they could inhibit the pathological process of Aβ, effectively protect neurons from Aβ-induced apoptosis, and maintain cognitive ability in AD mice (Huang et al., 2023). In addition, a class of multifunctional nanoparticles for externally controlled targeted delivery and release of drugs, called magnetoelectric nanoparticles, has recently been discovered (Pardo et al., 2021). Jongpil Kim and Youngeun Kwon have shown that electromagnetized gold nanoparticles (AuNPs) promote adult hippocampal neurogenesis, which improves cognitive function and memory consolidation in aged mice (Chang et al., 2021). Thus, increased efficiency of drug delivery to target brain tissue may prevent and reverse the course of human neurological disorders.

## 6. Conclusions and perspectives

The BBB is essential for providing the proper environment to allow normal neurological function and to protect the CNS from injury. BBB dysfunction is a pathological feature of AD, and this phenomenon may occur early in disease progression. BBB dysfunction may exacerbate Aβ production deposition, tau protein hyperphosphorylation, and neuroinflammation. In turn, these AD-related pathologies can exacerbate BBB disruption. However, it is not clear which of the two is the triggering factor. Currently, despite these explorations, the mechanisms regulating altered BBB function in AD patients remain unclear, which hinders further studies targeting functional regulation of the BBB to slow AD progression. The effects of complex multicellular structures and disease are difficult to accurately reproduce *in vitro*, and functional aspects of the BBB are not easily studied *in vitro*. Therefore, the use of *in vitro* models with excessive air leakage to study permeability may be problematic, and further studies are needed to improve the methods to detect the effects of BBB dysfunction on AD. While there are a variety of animal models available to aid in AD research, however, most models only exhibit some of the symptoms of AD. Future AD research could combine multiple model organism systems, using a simpler model organism (e.g., Drosophila) as an initial BBB test, which would help us narrow down the genes involved in the etiology of AD, and then go deeper in more complex model organisms (e.g., zebrafish, mice, or even non-human primates). High-throughput histological techniques and spatial proteomics, which have emerged in recent years, will help to provide new insights into the cellular and molecular mechanisms of BBB transporter function. Furthermore, to date, therapeutic efforts for neurological lesions remain heavily invested in the direct protection of axonal integrity and synaptic and overall neuronal health, with less research on BBB composition and maintenance of stability and integrity. It is hoped that an in-depth understanding of how the BBB affects the early stages of AD will be conducive to the identification of new effective targets for preventing or repairing the BBB, thereby slowing or halting the progression of AD. Most notably, even strategies to improve BBB function cannot overcome Aβ and tau. Therefore, unless specific mechanisms by which Aβ and tau affect the BBB become targets, the effects of Aβ and tau on the BBB will remain.

## Author contributions

YC: Writing – original draft, Writing – review & editing. YH: Writing – original draft, Writing – review & editing. JH: Writing – review & editing. WW: Writing – review & editing. FC: Funding acquisition, Writing – review & editing.

## Figures and Tables

**Table:** 

AD	Alzheimer’s disease
Aβ	Amyloid-beta
BBB	Blood–brain barrier
ApoE	Apolipoprotein E
CNS	Central nervous system
BEC	Brain endothelial cell
TJ	Tight junction
ZO-1	Zonula occludens-1
DCE-MRI	Dynamic contrast-enhanced magnetic resonance imaging
SWI	Susceptibility weighted imaging
CSVD	Cerebral small-vessel disease
^18^FDG-PET	^18^F-fluoro-2-deoxy-D-glucose positron emission tomography
MCI	Mild cognitive impairment
SUVr	Standardized uptake value ratio
DG	Dentate gyrus
CA1	Cornu ammonis
P-gp	P-glycoprotein
Alb	Albumin
Q-Alb	Albumin quotient
PDGFRβ	Platelet-derived growth factor receptor beta
FAD	Familial AD
EOAD	Early-onset AD
ELISA	Enzyme-linked immunosorbent assay
MRI	Magnetic resonance imaging
APP	Amyloid precursor protein
PS1	Presenilin-1
PS2	Presenilin-2
VEGFA	Vascular endothelial growth factor A
SERPINE1	Serpin family E member 1
PLAU	Urokinase-type plasminogen activator
MAPT	Microtubule-associated protein tau
NVUs	Neurovascular units
LRP1	Lipoprotein receptor-related protein-1
RAGE	Receptor for advanced glycation end products
ATP	Adenosine triphosphate
CAA	Cerebral amyloid angiopathy
Wnt	Wingless and int-1
β-catenin	Beta-catenin
GLUT1	Glucose transporter 1
NFTs	Neurofibrillary tangles
P-tau	Phosphorylated tau
PHF	Paired helix filaments
PB-MoM	Peripheral blood monocyte-derived macrophages
IL-1β	Interleukin-1beta
TNF-α	Tumor necrosis factor-alpha
IL-6	Interleukin-6
PRRs	Pattern recognition receptors
MMP9	Matrix metalloprotease 9
ALK	Activin receptor-like kinase
SMAD	Small mothers against decapentaplegic
NF-κB	Nuclear transcription factor-kappa B
Ca^2+^	Calcium ion
IL-17	Interleukin-17
Th1	T-helper 1
Th17	T-helper 17
CypA	Cyclophilin A
GFAP	Glial fibrillary acidic protein
GRP78	78-kDa glucose-regulated protein
IgG	Immunoglobulin G
COX-2	Cyclooxygenase-2
SAMP8	Accelerated senescence prone 8
SAMR1	Accelerated senescence resistant 1
snRNA-seq	Single-nucleus RNA-sequencing
Cx43	Connexin 43
Cdh5	Cadherin-5
NCI	No cognitive impairment
ECI	Early cognitive impairment
ARBs	Angiotensin receptor blockers
APC	Activated protein C
Ras	Renin angiotensin system
Rac1	Ras related C3 botulinum toxin substrate
iPSC	Induced pluripotent stem cell
FMT	Fecal microbiota transplantation
PICALM	Phosphatidylinositol-binding clathrin assembly protein
NPs	Nanoparticles
CMT	Carrier-mediated endocytosis
RMT	Receptor mediated endocytosis
Mg^2+^	Magnesium ion
CypD	Cyclophilin D
mPTP	Mitochondrial permeability transition pore
ROS	Reactive oxygen species

## References

[ref1] Akbari E. Asemi Z. Daneshvar Kakhaki R. Bahmani F. Kouchaki E. Tamtaji O. R.. (2016). Effect of probiotic supplementation on cognitive function and metabolic status in Alzheimer’s disease: a randomized, double-blind and controlled trial. Front. Aging Neurosci. 8:256. doi: 10.3389/fnagi.2016.00256 27891089 PMC5105117

[ref2] Alata W. Ye Y. St-Amour I. Vandal M. Calon F. (2015). Human apolipoprotein E varepsilon4 expression impairs cerebral vascularization and blood-brain barrier function in mice. J. Cereb. Blood Flow Metab. 35, 86–94. doi: 10.1038/jcbfm.2014.172 25335802 PMC4296574

[ref3] Allison Bosworth C. G. Chakhoyan A. Sagare A. P. Nelson A. R. Wang Y. Kisler K.. (2023). Molecular signature and functional properties of human pluripotent stem cell-derived brain pericytes. bioRxiv. doi: 10.1101/2023.06.26.546577

[ref4] Aslam M. Ahmad N. Srivastava R. Hemmer B. (2012). TNF-alpha induced NFkappaB signaling and p65 (RelA) overexpression repress Cldn5 promoter in mouse brain endothelial cells. Cytokine 57, 269–275. doi: 10.1016/j.cyto.2011.10.016, PMID: 22138107

[ref5] Atwal J. K. Chen Y. Chiu C. Mortensen D. L. Meilandt W. J. Liu Y.. (2011). A therapeutic antibody targeting BACE1 inhibits amyloid-beta production *in vivo*. Sci. Transl. Med. 3:84ra43. doi: 10.1126/scitranslmed.3002254 21613622

[ref6] Author (2022). Alzheimer’s disease facts and figures. Alzheimers Dement. 18, 700–789. doi: 10.1002/alz.12638 35289055

[ref7] Bell R. D. Winkler E. A. Singh I. Sagare A. P. Deane R. Wu Z.. (2012). Apolipoprotein E controls cerebrovascular integrity via cyclophilin A. Nature 485, 512–516. doi: 10.1038/nature11087 22622580 PMC4047116

[ref8] Bennett R. E. Robbins A. B. Hu M. Cao X. Betensky R. A. Clark T.. (2018). Tau induces blood vessel abnormalities and angiogenesis-related gene expression in P301L transgenic mice and human Alzheimer’s disease. Proc. Natl. Acad. Sci. U. S. A. 115, E1289–E1298. doi: 10.1073/pnas.1710329115 29358399 PMC5819390

[ref9] Bernal J. Valdes-Hernandez M. D. C. Escudero J. Heye A. K. Sakka E. Armitage P. A.. (2021). A four-dimensional computational model of dynamic contrast-enhanced magnetic resonance imaging measurement of subtle blood-brain barrier leakage. NeuroImage 230:117786. doi: 10.1016/j.neuroimage.2021.117786 33497771 PMC8065875

[ref10] Bettcher B. M. Tansey M. G. Dorothee G. Heneka M. T. (2021). Peripheral and central immune system crosstalk in Alzheimer disease – a research prospectus. Nat. Rev. Neurol. 17, 689–701. doi: 10.1038/s41582-021-00549-x 34522039 PMC8439173

[ref11] Bhattarai P. Thomas A. K. Zhang Y. Kizil C. (2017). The effects of aging on amyloid-beta42-induced neurodegeneration and regeneration in adult zebrafish brain. Neurogenesis 4:e1322666. doi: 10.1080/23262133.2017.1322666 28656156 PMC5477701

[ref12] Bolos M. Llorens-Martin M. Jurado-Arjona J. Hernandez F. Rabano A. Avila J. (2016). Direct evidence of internalization of tau by microglia *in vitro* and *in vivo*. J. Alzheimers Dis. 50, 77–87. doi: 10.3233/JAD-150704 26638867

[ref13] Bowman G. L. Kaye J. A. Moore M. Waichunas D. Carlson N. E. Quinn J. F. (2007). Blood-brain barrier impairment in Alzheimer disease: stability and functional significance. Neurology 68, 1809–1814. doi: 10.1212/01.wnl.0000262031.18018.1a 17515542 PMC2668699

[ref14] Braganza O. Bedner P. Huttmann K. von Staden E. Friedman A. Seifert G.. (2012). Albumin is taken up by hippocampal NG2 cells and astrocytes and decreases gap junction coupling. Epilepsia 53, 1898–1906. doi: 10.1111/j.1528-1167.2012.03665.x 22967085 PMC3651829

[ref15] Braniste V. Al-Asmakh M. Kowal C. Anuar F. Abbaspour A. Toth M.. (2014). The gut microbiota influences blood-brain barrier permeability in mice. Sci. Transl. Med. 6:263ra158. doi: 10.1126/scitranslmed.3009759 PMC4396848 25411471

[ref16] Bray N. (2015). Biologics: Transferrin’ bispecific antibodies across the blood-brain barrier. Nat. Rev. Drug Discov. 14, 14–15. doi: 10.1038/nrd4522 25503335

[ref17] Breijyeh Z. Karaman R. (2020). Comprehensive review on Alzheimer’s disease: causes and treatment. Molecules 25:5789. doi: 10.3390/molecules25245789 33302541 PMC7764106

[ref18] Brundel M. Heringa S. M. de Bresser J. Koek H. L. Zwanenburg J. J. Jaap Kappelle L.. (2012). High prevalence of cerebral microbleeds at 7Tesla MRI in patients with early Alzheimer’s disease. J. Alzheimers Dis. 31, 259–263. doi: 10.3233/JAD-2012-120364 22531417

[ref19] Cai Z. Liu N. Wang C. Qin B. Zhou Y. Xiao M.. (2016). Role of RAGE in Alzheimer’s disease. Cell. Mol. Neurobiol. 36, 483–495. doi: 10.1007/s10571-015-0233-3 26175217 PMC11482350

[ref20] Cao Y. Xu H. Zhu Y. Shi M. J. Wei L. Zhang J.. (2019). ADAMTS13 maintains cerebrovascular integrity to ameliorate Alzheimer-like pathology. PLoS Biol. 17:e3000313. doi: 10.1371/journal.pbio.3000313 31185010 PMC6588259

[ref21] Carlson M. L. DiGiacomo P. S. Fan A. P. Goubran M. Khalighi M. M. Chao S. Z.. (2020). Simultaneous FDG-PET/MRI detects hippocampal subfield metabolic differences in AD/MCI. Sci. Rep. 10:12064. doi: 10.1038/s41598-020-69065-0 32694602 PMC7374580

[ref22] Carrano A. Hoozemans J. J. van der Vies S. M. van Horssen J. de Vries H. E. Rozemuller A. J. (2012). Neuroinflammation and blood-brain barrier changes in capillary amyloid angiopathy. Neurodegener Dis 10, 329–331. doi: 10.1159/000334916 22301467

[ref23] Chang Y. Cho B. Lee E. Kim J. Yoo J. Sung J. S.. (2021). Electromagnetized gold nanoparticles improve neurogenesis and cognition in the aged brain. Biomaterials 278:121157. doi: 10.1016/j.biomaterials.2021.121157 34601195

[ref24] Chen Y. H. Lin R. R. Tao Q. Q. (2021). The role of P2X7R in neuroinflammation and implications in Alzheimer’s disease. Life Sci. 271:119187. doi: 10.1016/j.lfs.2021.119187 33577858

[ref25] Chen Z. L. Strickland S. (1997). Neuronal death in the hippocampus is promoted by plasmin-catalyzed degradation of laminin. Cells 91, 917–925. doi: 10.1016/s0092-8674(00)80483-3 9428515

[ref26] Cheng Z. Dai L. Wu Y. Cao Y. Chai X. Wang P.. (2023). Correlation of blood-brain barrier leakage with cerebral small vessel disease including cerebral microbleeds in Alzheimer’s disease. Front. Neurol. 14:1077860. doi: 10.3389/fneur.2023.1077860 36873442 PMC9978776

[ref27] Cisbani G. Rivest S. (2021). Targeting innate immunity to protect and cure Alzheimer’s disease: opportunities and pitfalls. Mol. Psychiatry 26, 5504–5515. doi: 10.1038/s41380-021-01083-4 33854189

[ref28] Crespo-Biel N. Theunis C. Van Leuven F. (2012). Protein tau: prime cause of synaptic and neuronal degeneration in Alzheimer’s disease. Int. J. Alzheimers Dis. 2012:251426. doi: 10.1155/2012/251426 22720188 PMC3376502

[ref29] de Leeuw S. M. Kirschner A. W. T. Lindner K. Rust R. Budny V. Wolski W. E.. (2022). APOE2, E3, and E4 differentially modulate cellular homeostasis, cholesterol metabolism, and inflammatory response in isogenic iPSC-derived astrocytes. Stem Cell Rep. 17, 110–126. doi: 10.1016/j.stemcr.2021.11.007 PMC8758949 34919811

[ref30] Deane R. Singh I. Sagare A. P. Bell R. D. Ross N. T. LaRue B.. (2012). A multimodal RAGE-specific inhibitor reduces amyloid beta-mediated brain disorder in a mouse model of Alzheimer disease. J. Clin. Invest. 122, 1377–1392. doi: 10.1172/JCI58642 22406537 PMC3314449

[ref31] Deo A. K. Borson S. Link J. M. Domino K. Eary J. F. Ke B.. (2014). Activity of P-glycoprotein, a beta-amyloid transporter at the blood-brain barrier, is compromised in patients with mild Alzheimer disease. J. Nucl. Med. 55, 1106–1111. doi: 10.2967/jnumed.113.130161 24842892 PMC4691246

[ref32] Deshwar A. R. Cytrynbaum C. Murthy H. Zon J. Chitayat D. Volpatti J.. (2023). Variants in CLDN5 cause a syndrome characterized by seizures, microcephaly and brain calcifications. Brain 146, 2285–2297. doi: 10.1093/brain/awac461 36477332

[ref33] DeTure M. A. Dickson D. W. (2019). The neuropathological diagnosis of Alzheimer’s disease. Mol. Neurodegener. 14:32. doi: 10.1186/s13024-019-0333-5 31375134 PMC6679484

[ref34] Dickie B. R. Boutin H. Parker G. J. M. Parkes L. M. (2021). Alzheimer’s disease pathology is associated with earlier alterations to blood-brain barrier water permeability compared with healthy ageing in TgF344-AD rats. NMR Biomed. 34:e4510. doi: 10.1002/nbm.4510 33723901 PMC11475392

[ref35] Edara V. V. Nooka S. Proulx J. Stacy S. Ghorpade A. Borgmann K. (2020). beta-catenin regulates wound healing and IL-6 expression in activated human astrocytes. Biomedicine 8:479. doi: 10.3390/biomedicines8110479 PMC7694627 33171974

[ref36] Elahy M. Jackaman C. Mamo J. C. Lam V. Dhaliwal S. S. Giles C.. (2015). Blood-brain barrier dysfunction developed during normal aging is associated with inflammation and loss of tight junctions but not with leukocyte recruitment. Immun. Ageing 12:2. doi: 10.1186/s12979-015-0029-9 25784952 PMC4362825

[ref37] Erdo F. Krajcsi P. (2019). Age-related functional and expressional changes in efflux pathways at the blood-brain barrier. Front. Aging Neurosci. 11:196. doi: 10.3389/fnagi.2019.00196 31417399 PMC6682691

[ref38] Erickson M. A. Banks W. A. (2013). Blood-brain barrier dysfunction as a cause and consequence of Alzheimer’s disease. J. Cereb. Blood Flow Metab. 33, 1500–1513. doi: 10.1038/jcbfm.2013.135 23921899 PMC3790938

[ref39] Faal T. Phan D. T. T. Davtyan H. Scarfone V. M. Varady E. Blurton-Jones M.. (2019). Induction of mesoderm and neural crest-derived Pericytes from human pluripotent stem cells to study blood-brain barrier interactions. Stem Cell Rep. 12, 451–460. doi: 10.1016/j.stemcr.2019.01.005 PMC6409424 30745035

[ref40] Farrall A. J. Wardlaw J. M. (2009). Blood-brain barrier: ageing and microvascular disease--systematic review and meta-analysis. Neurobiol. Aging 30, 337–352. doi: 10.1016/j.neurobiolaging.2007.07.015 17869382

[ref41] Fiala M. Liu Q. N. Sayre J. Pop V. Brahmandam V. Graves M. C.. (2002). Cyclooxygenase-2-positive macrophages infiltrate the Alzheimer’s disease brain and damage the blood-brain barrier. Eur. J. Clin. Investig. 32, 360–371. doi: 10.1046/j.1365-2362.2002.00994.x 12027877

[ref42] Frozza R. L. Lourenco M. V. De Felice F. G. (2018). Challenges for Alzheimer’s disease therapy: insights from novel mechanisms beyond memory defects. Front. Neurosci. 12:37. doi: 10.3389/fnins.2018.00037 29467605 PMC5808215

[ref43] Giannoni P. Arango-Lievano M. Neves I. D. Rousset M. C. Baranger K. Rivera S.. (2016). Cerebrovascular pathology during the progression of experimental Alzheimer’s disease. Neurobiol. Dis. 88, 107–117. doi: 10.1016/j.nbd.2016.01.001 26774030

[ref44] Griffin J. H. Zlokovic B. V. Mosnier L. O. (2015). Activated protein C: biased for translation. Blood 125, 2898–2907. doi: 10.1182/blood-2015-02-355974 25824691 PMC4424414

[ref45] Halliday M. R. Rege S. V. Ma Q. Zhao Z. Miller C. A. Winkler E. A.. (2016). Accelerated pericyte degeneration and blood-brain barrier breakdown in apolipoprotein E4 carriers with Alzheimer’s disease. J. Cereb. Blood Flow Metab. 36, 216–227. doi: 10.1038/jcbfm.2015.44 25757756 PMC4758554

[ref46] Hartz A. M. Bauer B. Soldner E. L. Wolf A. Boy S. Backhaus R.. (2012). Amyloid-beta contributes to blood-brain barrier leakage in transgenic human amyloid precursor protein mice and in humans with cerebral amyloid angiopathy. Stroke 43, 514–523. doi: 10.1161/STROKEAHA.111.627562 22116809 PMC5761312

[ref47] Huang D. Wang Q. Cao Y. Yang H. Li M. Wu F.. (2023). Multiscale NIR-II imaging-guided brain-targeted drug delivery using engineered cell membrane Nanoformulation for Alzheimer’s disease therapy. ACS Nano. 17, 5033–5046. doi: 10.1021/acsnano.2c12840 36867454

[ref48] Jackson R. J. Meltzer J. C. Nguyen H. Commins C. Bennett R. E. Hudry E.. (2022). APOE4 derived from astrocytes leads to blood-brain barrier impairment. Brain 145, 3582–3593. doi: 10.1093/brain/awab478 34957486 PMC9586546

[ref49] John G. R. Lee S. C. Brosnan C. F. (2003). Cytokines: powerful regulators of glial cell activation. Neuroscientist 9, 10–22. doi: 10.1177/1073858402239587 12580336

[ref50] Jones A. R. Shusta E. V. (2007). Blood-brain barrier transport of therapeutics via receptor-mediation. Pharm. Res. 24, 1759–1771. doi: 10.1007/s11095-007-9379-0 17619996 PMC2685177

[ref51] Kang S. G. Shinojima N. Hossain A. Gumin J. Yong R. L. Colman H.. (2010). Isolation and perivascular localization of mesenchymal stem cells from mouse brain. Neurosurgery 67, 711–720. doi: 10.1227/01.NEU.0000377859.06219.78 20651630 PMC3644957

[ref52] Kato T. Inui Y. Nakamura A. Ito K. (2016). Brain fluorodeoxyglucose (FDG) PET in dementia. Ageing Res. Rev. 30, 73–84. doi: 10.1016/j.arr.2016.02.003 26876244

[ref53] Kim N. Jeon S. H. Ju I. G. Gee M. S. Do J. Oh M. S.. (2021). Transplantation of gut microbiota derived from Alzheimer’s disease mouse model impairs memory function and neurogenesis in C57BL/6 mice. Brain Behav. Immun. 98, 357–365. doi: 10.1016/j.bbi.2021.09.002 34500036

[ref54] Kisler K. Sagare A. P. Lazic D. Bazzi S. Lawson E. Hsu C. J.. (2023). Anti-malaria drug artesunate prevents development of amyloid-beta pathology in mice by upregulating PICALM at the blood-brain barrier. Mol. Neurodegener. 18:7. doi: 10.1186/s13024-023-00597-5 36707892 PMC9883925

[ref55] Kolarova M. Garcia-Sierra F. Bartos A. Ricny J. Ripova D. (2012). Structure and pathology of tau protein in Alzheimer disease. Int. J. Alzheimers Dis. 2012:731526. doi: 10.1155/2012/731526 22690349 PMC3368361

[ref56] Kurz C. Walker L. Rauchmann B. S. Perneczky R. (2022). Dysfunction of the blood-brain barrier in Alzheimer’s disease: evidence from human studies. Neuropathol. Appl. Neurobiol. 48:e12782. doi: 10.1111/nan.12782 34823269

[ref57] Langen U. H. Ayloo S. Gu C. (2019). Development and cell biology of the blood-brain barrier. Annu. Rev. Cell Dev. Biol. 35, 591–613. doi: 10.1146/annurev-cellbio-100617-062608 31299172 PMC8934576

[ref58] Lazic D. Sagare A. P. Nikolakopoulou A. M. Griffin J. H. Vassar R. Zlokovic B. V. (2019). 3K3A-activated protein C blocks amyloidogenic BACE1 pathway and improves functional outcome in mice. J. Exp. Med. 216, 279–293. doi: 10.1084/jem.20181035 30647119 PMC6363429

[ref59] Lee H. J. Lee K. E. Kim J. K. Kim D. H. (2019). Suppression of gut dysbiosis by *Bifidobacterium longum* alleviates cognitive decline in 5XFAD transgenic and aged mice. Sci. Rep. 9:11814. doi: 10.1038/s41598-019-48342-7 31413350 PMC6694197

[ref60] Lee D. C. Rizer J. Selenica M. L. Reid P. Kraft C. Johnson A.. (2010). LPS- induced inflammation exacerbates phospho-tau pathology in rTg4510 mice. J. Neuroinflammation 7:56. doi: 10.1186/1742-2094-7-56 20846376 PMC2949628

[ref61] LeVine S. M. (2016). Albumin and multiple sclerosis. BMC Neurol. 16:47. doi: 10.1186/s12883-016-0564-9 27067000 PMC4828783

[ref62] Li X. Quan M. Wei Y. Wang W. Xu L. Wang Q.. (2023). Critical thinking of Alzheimer’s transgenic mouse model: current research and future perspective. Sci. China Life Sci. 1–44. doi: 10.1007/s11427-022-2357-x 37480469

[ref63] Liebner S. Dijkhuizen R. M. Reiss Y. Plate K. H. Agalliu D. Constantin G. (2018). Functional morphology of the blood-brain barrier in health and disease. Acta Neuropathol. 135, 311–336. doi: 10.1007/s00401-018-1815-1 29411111 PMC6781630

[ref64] Lin Y. F. Smith A. V. Aspelund T. Betensky R. A. Smoller J. W. Gudnason V.. (2019). Genetic overlap between vascular pathologies and Alzheimer’s dementia and potential causal mechanisms. Alzheimers Dement. 15, 65–75. doi: 10.1016/j.jalz.2018.08.002 30240575 PMC6435328

[ref65] Liu F. Li B. Tung E. J. Grundke-Iqbal I. Iqbal K. Gong C. X. (2007). Site-specific effects of tau phosphorylation on its microtubule assembly activity and self-aggregation. Eur. J. Neurosci. 26, 3429–3436. doi: 10.1111/j.1460-9568.2007.05955.x 18052981 PMC2262108

[ref66] Maiuolo J. Gliozzi M. Musolino V. Scicchitano M. Carresi C. Scarano F.. (2018). The “frail” brain blood barrier in neurodegenerative diseases: role of early disruption of endothelial cell-to-cell connections. Int. J. Mol. Sci. 19:2693. doi: 10.3390/ijms19092693 30201915 PMC6164949

[ref67] Majerova P. Michalicova A. Cente M. Hanes J. Vegh J. Kittel A.. (2019). Trafficking of immune cells across the blood-brain barrier is modulated by neurofibrillary pathology in tauopathies. PLoS One 14:e0217216. doi: 10.1371/journal.pone.0217216 31120951 PMC6532920

[ref68] Marques F. Sousa J. C. Sousa N. Palha J. A. (2013). Blood-brain-barriers in aging and in Alzheimer’s disease. Mol. Neurodegener. 8:38. doi: 10.1186/1750-1326-8-38 24148264 PMC4015275

[ref69] McGeer P. L. McGeer E. G. (1995). The inflammatory response system of brain: implications for therapy of Alzheimer and other neurodegenerative diseases. Brain Res. Brain Res. Rev. 21, 195–218. doi: 10.1016/0165-0173(95)00011-9 8866675

[ref70] Miners J. S. Kehoe P. G. Love S. Zetterberg H. Blennow K. (2019). CSF evidence of pericyte damage in Alzheimer’s disease is associated with markers of blood-brain barrier dysfunction and disease pathology. Alzheimers Res. Ther. 11:81. doi: 10.1186/s13195-019-0534-8 31521199 PMC6745071

[ref71] Moir R. D. Tanzi R. E. (2005). LRP-mediated clearance of Abeta is inhibited by KPI-containing isoforms of APP. Curr. Alzheimer Res. 2, 269–273. doi: 10.2174/1567205053585918 15974929

[ref72] Montagne A. Barnes S. R. Sweeney M. D. Halliday M. R. Sagare A. P. Zhao Z.. (2015). Blood-brain barrier breakdown in the aging human hippocampus. Neuron 85, 296–302. doi: 10.1016/j.neuron.2014.12.032 25611508 PMC4350773

[ref73] Montagne A. Nation D. A. Sagare A. P. Barisano G. Sweeney M. D. Chakhoyan A.. (2020). APOE4 leads to blood-brain barrier dysfunction predicting cognitive decline. Nature 581, 71–76. doi: 10.1038/s41586-020-2247-3 32376954 PMC7250000

[ref74] Montagne A. Nikolakopoulou A. M. Huuskonen M. T. Sagare A. P. Lawson E. J. Lazic D.. (2021). APOE4 accelerates advanced-stage vascular and neurodegenerative disorder in old Alzheimer’s mice via cyclophilin a independently of amyloid-beta. Nat. Aging 1, 506–520. doi: 10.1038/s43587-021-00073-z 35291561 PMC8920485

[ref75] Montagne A. Zhao Z. Zlokovic B. V. (2017). Alzheimer’s disease: a matter of blood-brain barrier dysfunction? J. Exp. Med. 214, 3151–3169. doi: 10.1084/jem.20171406 29061693 PMC5679168

[ref76] Munji R. N. Daneman R. (2020). Unexpected amount of blood-borne protein enters the young brain. Nature 583, 362–363. doi: 10.1038/d41586-020-01791-x 32661413

[ref77] Musaeus C. S. Gleerup H. S. Hogh P. Waldemar G. Hasselbalch S. G. Simonsen A. H. (2020). Cerebrospinal fluid/plasma albumin ratio as a biomarker for blood-brain barrier impairment across neurodegenerative dementias. J. Alzheimers Dis. 75, 429–436. doi: 10.3233/JAD-200168 32280104

[ref78] Nation D. A. Sweeney M. D. Montagne A. Sagare A. P. D’Orazio L. M. Pachicano M.. (2019). Blood-brain barrier breakdown is an early biomarker of human cognitive dysfunction. Nat. Med. 25, 270–276. doi: 10.1038/s41591-018-0297-y 30643288 PMC6367058

[ref79] Nelson A. R. Sweeney M. D. Sagare A. P. Zlokovic B. V. (2016). Neurovascular dysfunction and neurodegeneration in dementia and Alzheimer’s disease. Biochim. Biophys. Acta 1862, 887–900. doi: 10.1016/j.bbadis.2015.12.016 26705676 PMC4821735

[ref80] Niewoehner J. Bohrmann B. Collin L. Urich E. Sade H. Maier P.. (2014). Increased brain penetration and potency of a therapeutic antibody using a monovalent molecular shuttle. Neuron 81, 49–60. doi: 10.1016/j.neuron.2013.10.061 24411731

[ref81] Nikolakopoulou A. M. Wang Y. Ma Q. Sagare A. P. Montagne A. Huuskonen M. T.. (2021). Endothelial LRP1 protects against neurodegeneration by blocking cyclophilin a. J. Exp. Med. 218:2207. doi: 10.1084/jem.20202207 PMC7863706 33533918

[ref82] Nishitsuji K. Hosono T. Nakamura T. Bu G. Michikawa M. (2011). Apolipoprotein E regulates the integrity of tight junctions in an isoform-dependent manner in an in vitro blood-brain barrier model. J. Biol. Chem. 286, 17536–17542. doi: 10.1074/jbc.M111.225532 21471207 PMC3093828

[ref83] Ohene Y. Harrison I. F. Evans P. G. Thomas D. L. Lythgoe M. F. Wells J. A. (2021). Increased blood-brain barrier permeability to water in the aging brain detected using noninvasive multi-TE ASL MRI. Magn. Reson. Med. 85, 326–333. doi: 10.1002/mrm.28496 32910547 PMC8432141

[ref84] Olsson B. Lautner R. Andreasson U. Ohrfelt A. Portelius E. Bjerke M.. (2016). CSF and blood biomarkers for the diagnosis of Alzheimer’s disease: a systematic review and meta-analysis. Lancet Neurol. 15, 673–684. doi: 10.1016/S1474-4422(16)00070-3 27068280

[ref85] Pardo M. Roberts E. R. Pimentel K. Yildirim Y. A. Navarrete B. Wang P.. (2021). Size-dependent intranasal administration of magnetoelectric nanoparticles for targeted brain localization. Nanomedicine 32:102337. doi: 10.1016/j.nano.2020.102337 33197627

[ref86] Pardridge W. M. (2015). Targeted delivery of protein and gene medicines through the blood-brain barrier. Clin. Pharmacol. Ther. 97, 347–361. doi: 10.1002/cpt.18 25669455

[ref87] Parker A. Fonseca S. Carding S. R. (2020). Gut microbes and metabolites as modulators of blood-brain barrier integrity and brain health. Gut Microbes 11, 135–157. doi: 10.1080/19490976.2019.1638722 31368397 PMC7053956

[ref88] Parodi-Rullan R. M. Javadov S. Fossati S. (2021). Dissecting the crosstalk between endothelial mitochondrial damage, vascular inflammation, and neurodegeneration in cerebral amyloid Angiopathy and Alzheimer’s disease. Cells 10:2903. doi: 10.3390/cells10112903 34831125 PMC8616424

[ref89] Paul G. Ozen I. Christophersen N. S. Reinbothe T. Bengzon J. Visse E.. (2012). The adult human brain harbors multipotent perivascular mesenchymal stem cells. PLoS One 7:e35577. doi: 10.1371/journal.pone.0035577 22523602 PMC3327668

[ref90] Paul J. Strickland S. Melchor J. P. (2007). Fibrin deposition accelerates neurovascular damage and neuroinflammation in mouse models of Alzheimer’s disease. J. Exp. Med. 204, 1999–2008. doi: 10.1084/jem.20070304 17664291 PMC2118680

[ref91] Pelegri C. Canudas A. M. del Valle J. Casadesus G. Smith M. A. Camins A.. (2007). Increased permeability of blood-brain barrier on the hippocampus of a murine model of senescence. Mech. Ageing Dev. 128, 522–528. doi: 10.1016/j.mad.2007.07.002 17697702

[ref92] Propson N. E. Roy E. R. Litvinchuk A. Kohl J. Zheng H. (2021). Endothelial C3a receptor mediates vascular inflammation and blood-brain barrier permeability during aging. J. Clin. Invest. 131:966. doi: 10.1172/JCI140966 PMC7773352 32990682

[ref93] Ren Q. Chen H. Chen Y. Song Z. Ouyang S. Lian S.. (2023). Imine-linked covalent organic framework modulates oxidative stress in Alzheimer’s disease. ACS Appl. Mater. Interfaces 15, 4947–4958. doi: 10.1021/acsami.2c19839 36651694

[ref94] Riphagen J. M. Ramakers I. Freeze W. M. Pagen L. H. G. Hanseeuw B. J. Verbeek M. M.. (2020). Linking APOE-epsilon4, blood-brain barrier dysfunction, and inflammation to Alzheimer’s pathology. Neurobiol. Aging 85, 96–103. doi: 10.1016/j.neurobiolaging.2019.09.020 31733942

[ref95] Rodriguez-Ortiz C. J. Thorwald M. A. Rodriguez R. Mejias-Ortega M. Kieu Z. Maitra N.. (2023). Angiotensin receptor blockade with olmesartan alleviates brain pathology in obese OLETF rats. Clin. Exp. Pharmacol. Physiol. 50, 228–237. doi: 10.1111/1440-1681.13738 36398458 PMC9898104

[ref96] Ryu J. K. McLarnon J. G. (2009). A leaky blood-brain barrier, fibrinogen infiltration and microglial reactivity in inflamed Alzheimer’s disease brain. J. Cell. Mol. Med. 13, 2911–2925. doi: 10.1111/j.1582-4934.2008.00434.x 18657226 PMC4498946

[ref97] Sagare A. P. Sweeney M. D. Makshanoff J. Zlokovic B. V. (2015). Shedding of soluble platelet-derived growth factor receptor-beta from human brain pericytes. Neurosci. Lett. 607, 97–101. doi: 10.1016/j.neulet.2015.09.025 26407747 PMC4631673

[ref98] Shang J. Yamashita T. Tian F. Li X. Liu X. Shi X.. (2019). Chronic cerebral hypoperfusion alters amyloid-beta transport related proteins in the cortical blood vessels of Alzheimer’s disease model mouse. Brain Res. 1723:146379. doi: 10.1016/j.brainres.2019.146379 31415766

[ref99] Sharma H. S. Castellani R. J. Smith M. A. Sharma A. (2012). The blood-brain barrier in Alzheimer’s disease: novel therapeutic targets and nanodrug delivery. Int. Rev. Neurobiol. 102, 47–90. doi: 10.1016/B978-0-12-386986-9.00003-X 22748826

[ref100] Shibata M. Yamada S. Kumar S. R. Calero M. Bading J. Frangione B.. (2000). Clearance of Alzheimer’s amyloid-ss(1-40) peptide from brain by LDL receptor-related protein-1 at the blood-brain barrier. J. Clin. Invest. 106, 1489–1499. doi: 10.1172/JCI10498 11120756 PMC387254

[ref101] Shigemoto-Mogami Y. Hoshikawa K. Sato K. (2018). Activated microglia disrupt the blood-brain barrier and induce chemokines and cytokines in a rat in vitro model. Front. Cell. Neurosci. 12:494. doi: 10.3389/fncel.2018.00494 30618641 PMC6300509

[ref102] Simpson I. A. Chundu K. R. Davies-Hill T. Honer W. G. Davies P. (1994). Decreased concentrations of GLUT1 and GLUT3 glucose transporters in the brains of patients with Alzheimer’s disease. Ann. Neurol. 35, 546–551. doi: 10.1002/ana.410350507 8179300

[ref103] Skillback T. Delsing L. Synnergren J. Mattsson N. Janelidze S. Nagga K.. (2017). CSF/serum albumin ratio in dementias: a cross-sectional study on 1861 patients. Neurobiol. Aging 59, 1–9. doi: 10.1016/j.neurobiolaging.2017.06.028 28779628

[ref104] Slemmon J. R. Hughes C. M. Campbell G. A. Flood D. G. (1994). Increased levels of hemoglobin-derived and other peptides in Alzheimer’s disease cerebellum. J. Neurosci. 14, 2225–2235. doi: 10.1523/JNEUROSCI.14-04-02225.1994 7512635 PMC6577136

[ref105] Storck S. E. Hartz A. M. S. Bernard J. Wolf A. Kachlmeier A. Mahringer A.. (2018). The concerted amyloid-beta clearance of LRP1 and ABCB1/P-gp across the blood-brain barrier is linked by PICALM. Brain Behav. Immun. 73, 21–33. doi: 10.1016/j.bbi.2018.07.017 30041013 PMC7748946

[ref106] Sun J. Xu J. Ling Y. Wang F. Gong T. Yang C.. (2019). Fecal microbiota transplantation alleviated Alzheimer’s disease-like pathogenesis in APP/PS1 transgenic mice. Transl. Psychiatry 9:189. doi: 10.1038/s41398-019-0525-3 31383855 PMC6683152

[ref107] Sun Z. Zhao H. Fang D. Davis C. T. Shi D. S. Lei K.. (2022). Neuroinflammatory disease disrupts the blood-CNS barrier via crosstalk between proinflammatory and endothelial-to-mesenchymal-transition signaling. Neuron 110, 3106–3120.e7. doi: 10.1016/j.neuron.2022.07.015 35961320 PMC9547934

[ref108] Sweeney M. D. Sagare A. P. Pachicano M. Harrington M. G. Joe E. Chui H. C.. (2020). A novel sensitive assay for detection of a biomarker of pericyte injury in cerebrospinal fluid. Alzheimers Dement. 16, 821–830. doi: 10.1002/alz.12061 32301266 PMC7986963

[ref109] Sweeney M. D. Sagare A. P. Zlokovic B. V. (2018). Blood-brain barrier breakdown in Alzheimer disease and other neurodegenerative disorders. Nat. Rev. Neurol. 14, 133–150. doi: 10.1038/nrneurol.2017.188 29377008 PMC5829048

[ref110] Sweeney M. D. Zhao Z. Montagne A. Nelson A. R. Zlokovic B. V. (2019). Blood-brain barrier: from physiology to disease and Back. Physiol. Rev. 99, 21–78. doi: 10.1152/physrev.00050.2017 30280653 PMC6335099

[ref111] Szablewski L. (2021). Brain glucose transporters: role in pathogenesis and potential targets for the treatment of Alzheimer’s disease. Int. J. Mol. Sci. 22:8142. doi: 10.3390/ijms22158142 34360906 PMC8348194

[ref112] Tarasoff-Conway J. M. Carare R. O. Osorio R. S. Glodzik L. Butler T. Fieremans E.. (2015). Clearance systems in the brain-implications for Alzheimer disease. Nat. Rev. Neurol. 11, 457–470. doi: 10.1038/nrneurol.2015.119 26195256 PMC4694579

[ref113] Tiani K. A. Stover P. J. Field M. S. (2019). The role of brain barriers in maintaining brain vitamin levels. Annu. Rev. Nutr. 39, 147–173. doi: 10.1146/annurev-nutr-082018-124235 31150592 PMC11791776

[ref114] Tibbling G. Link H. Ohman S. (1977). Principles of albumin and IgG analyses in neurological disorders. I. Establishment of reference values. Scand. J. Clin. Lab. Invest. 37, 385–390. doi: 10.1080/00365517709091496 337459

[ref115] Ueno M. Akiguchi I. Yagi H. Naiki H. Fujibayashi Y. Kimura J.. (1993). Age-related changes in barrier function in mouse brain I. Accelerated age-related increase of brain transfer of serum albumin in accelerated senescence prone SAM-P/8 mice with deficits in learning and memory. Arch. Gerontol. Geriatr. 16, 233–248. doi: 10.1016/0167-4943(93)90035-g 15374337

[ref116] Uetani H. Hirai T. Hashimoto M. Ikeda M. Kitajima M. Sakamoto F.. (2013). Prevalence and topography of small hypointense foci suggesting microbleeds on 3T susceptibility-weighted imaging in various types of dementia. AJNR Am. J. Neuroradiol. 34, 984–989. doi: 10.3174/ajnr.A3332 23124636 PMC7964666

[ref117] Valdes A. M. Walter J. Segal E. Spector T. D. (2018). Role of the gut microbiota in nutrition and health. BMJ 361:k2179. doi: 10.1136/bmj.k2179 29899036 PMC6000740

[ref118] van de Haar H. J. Burgmans S. Jansen J. F. van Osch M. J. van Buchem M. A. Muller M.. (2016). Blood-brain barrier leakage in patients with early Alzheimer disease. Radiology 281, 527–535. doi: 10.1148/radiol.2016152244 27243267

[ref119] Verghese P. B. Castellano J. M. Holtzman D. M. (2011). Apolipoprotein E in Alzheimer’s disease and other neurological disorders. Lancet Neurol. 10, 241–252. doi: 10.1016/S1474-4422(10)70325-2 21349439 PMC3132088

[ref120] Wang Q. Huang X. Su Y. Yin G. Wang S. Yu B.. (2022). Activation of Wnt/beta-catenin pathway mitigates blood-brain barrier dysfunction in Alzheimer’s disease. Brain 145, 4474–4488. doi: 10.1093/brain/awac236 35788280 PMC9762951

[ref121] Winkler E. A. Nishida Y. Sagare A. P. Rege S. V. Bell R. D. Perlmutter D.. (2015). GLUT1 reductions exacerbate Alzheimer’s disease vasculo-neuronal dysfunction and degeneration. Nat. Neurosci. 18, 521–530. doi: 10.1038/nn.3966 25730668 PMC4734893

[ref122] Winkler E. A. Sengillo J. D. Sagare A. P. Zhao Z. Ma Q. Zuniga E.. (2014). Blood-spinal cord barrier disruption contributes to early motor-neuron degeneration in ALS-model mice. Proc. Natl. Acad. Sci. U. S. A. 111, E1035–E1042. doi: 10.1073/pnas.1401595111 24591593 PMC3964055

[ref123] Wisniewski H. M. Kozlowski P. B. (1982). Evidence for blood-brain barrier changes in senile dementia of the Alzheimer type (SDAT). Ann. N. Y. Acad. Sci. 396, 119–129. doi: 10.1111/j.1749-6632.1982.tb26848.x 6185032

[ref124] Yang J. Ran M. Li H. Lin Y. Ma K. Yang Y.. (2022). New insight into neurological degeneration: inflammatory cytokines and blood-brain barrier. Front. Mol. Neurosci. 15:1013933. doi: 10.3389/fnmol.2022.1013933 36353359 PMC9637688

[ref125] Yang F. Zhao K. Zhang X. Zhang J. Xu B. (2016). ATP induces disruption of tight junction proteins via IL-1 Beta-dependent MMP-9 activation of human blood-brain barrier in vitro. Neural Plast. 2016:8928530. doi: 10.1155/2016/8928530 27795859 PMC5067334

[ref126] Yu Y. J. Atwal J. K. Zhang Y. Tong R. K. Wildsmith K. R. Tan C.. (2014). Therapeutic bispecific antibodies cross the blood-brain barrier in nonhuman primates. Sci. Transl. Med. 6:261ra154. doi: 10.1126/scitranslmed.3009835 25378646

[ref127] Zenaro E. Piacentino G. Constantin G. (2017). The blood-brain barrier in Alzheimer’s disease. Neurobiol. Dis. 107, 41–56. doi: 10.1016/j.nbd.2016.07.007 27425887 PMC5600438

[ref128] Zenaro E. Pietronigro E. Della Bianca V. Piacentino G. Marongiu L. Budui S.. (2015). Neutrophils promote Alzheimer’s disease-like pathology and cognitive decline via LFA-1 integrin. Nat. Med. 21, 880–886. doi: 10.1038/nm.3913 26214837

[ref129] Zhan R. Meng X. Tian D. Xu J. Cui H. Yang J.. (2023). NAD(+) rescues aging-induced blood-brain barrier damage via the CX43-PARP1 axis. Neuron. 111, 1–16. doi: 10.1016/j.neuron.2023.08.010 37683629

[ref130] Zhang T. T. Li W. Meng G. Wang P. Liao W. (2016). Strategies for transporting nanoparticles across the blood-brain barrier. Biomater. Sci. 4, 219–229. doi: 10.1039/c5bm00383k 26646694

[ref131] Zhang Q. Song Q. Yu R. Wang A. Jiang G. Huang Y.. (2023). Nano-brake halts mitochondrial dysfunction Cascade to alleviate neuropathology and rescue Alzheimer’s cognitive deficits. Adv. Sci. 10:e2204596. doi: 10.1002/advs.202204596 PMC9982524 36703613

[ref132] Zhang G. S. Tian Y. Huang J. Y. Tao R. R. Liao M. H. Lu Y. M.. (2013). The gamma-secretase blocker DAPT reduces the permeability of the blood-brain barrier by decreasing the ubiquitination and degradation of occludin during permanent brain ischemia. CNS Neurosci. Ther. 19, 53–60. doi: 10.1111/cns.12032 23171401 PMC6493664

[ref133] Zhao Z. Nelson A. R. Betsholtz C. Zlokovic B. V. (2015). Establishment and dysfunction of the blood-brain barrier. Cells 163, 1064–1078. doi: 10.1016/j.cell.2015.10.067 PMC4655822 26590417

[ref134] Zhong X. Na Y. Yin S. Yan C. Gu J. Zhang N.. (2023). Cell membrane biomimetic nanoparticles with potential in treatment of Alzheimer’s disease. Molecules 28:2336. doi: 10.3390/molecules28052336 36903581 PMC10005336

[ref135] Zhou Y. Zhu F. Liu Y. Zheng M. Wang Y. Zhang D.. (2020). Blood-brain barrier-penetrating siRNA nanomedicine for Alzheimer’s disease therapy. Sci. Adv. 6:7031. doi: 10.1126/sciadv.abc7031 PMC7546706 33036977

[ref136] Zilka N. Stozicka Z. Kovac A. Pilipcinec E. Bugos O. Novak M. (2009). Human misfolded truncated tau protein promotes activation of microglia and leukocyte infiltration in the transgenic rat model of tauopathy. J. Neuroimmunol. 209, 16–25. doi: 10.1016/j.jneuroim.2009.01.013 19232747

[ref137] Zipser B. D. Johanson C. E. Gonzalez L. Berzin T. M. Tavares R. Hulette C. M.. (2007). Microvascular injury and blood-brain barrier leakage in Alzheimer’s disease. Neurobiol. Aging 28, 977–986. doi: 10.1016/j.neurobiolaging.2006.05.016 16782234

[ref138] Zlokovic B. V. (2008). The blood-brain barrier in health and chronic neurodegenerative disorders. Neuron 57, 178–201. doi: 10.1016/j.neuron.2008.01.003 18215617

[ref139] Zlokovic B. V. (2011). Neurovascular pathways to neurodegeneration in Alzheimer’s disease and other disorders. Nat. Rev. Neurosci. 12, 723–738. doi: 10.1038/nrn3114 22048062 PMC4036520

